# Inductively Expandable Supraparticles as Microscopic Force Generators for Remote Mechanical Actuation

**DOI:** 10.1002/adma.74932

**Published:** 2026-09-15

**Authors:** Leoni Luthardt, Stephan Müssig, Robert Luxenhofer, Karl Mandel

**Affiliations:** ^1^ Chair ‘Particle‐Based Materials Chemistry’ Section Materials Chemistry Department of Chemistry and Pharmacy Friedrich‐Alexander‐Universität Erlangen‐Nürnberg (FAU) Erlangen Germany; ^2^ Soft Matter Chemistry Department of Chemistry and Helsinki Institute of Sustainability Science Faculty of Science University of Helsinki Helsinki Finland

**Keywords:** blowing agents, induction heating, magnetic iron oxide nanoparticles, mechanical actuation, micron‐scale force generation, supraparticles

## Abstract

Generating strong mechanical forces at the microscale is central to emerging technologies like soft microrobotics and adaptive materials. However, delivering energy remotely and converting it into mechanical work within confined environments remains challenging. Magnetic nanoparticles enable rapid, contactless heat generation through induction heating under alternating magnetic fields, yet this is typically exploited only for thermal effects. Here, we introduce micrometer‐scaled supraparticle additives that convert induction‐generated heat directly into mechanical work through confined expansion. These supraparticles are fabricated by spray‐drying magnetic nanoparticles with functional blowing agents, enabling actuation by rapid gas release using azodicarbonamide or volumetric expansion via vaporization‐induced volume expansion within a superabsorbent poly(acrylamide‐*co*‐acrylic acid) network. Upon magnetic excitation, heating generates pressure within the supraparticles, yielding power densities up to 5 kW kg^−1^ and lifting capabilities of up to 25 000 times their own mass, exceeding the typical limit of conventional materials (∼10 000x). Actuation is achieved with heating times of ≤5 s and total energy consumption below 3 Wh, remaining reproducible over multiple actuation cycles without failures. Generated stresses are sufficient to disrupt mechanically stable matrices, including rigid epoxies. These results establish supraparticles as versatile platforms for remotely powered mechanical actuation, enabling localized stress generation and material disruption in interactive materials.

## Graphical abstract

This study demonstrates the combination of magnetic nanoparticles and functional blowing agents into supraparticles. Upon induction heating, they actuate via rapid gas generation or extensive volume expansion. These supraparticles lift up to 25 000 times their own mass and rapidly open cured epoxy resins, providing a versatile strategy for fast micron‐scale pressure actuation.

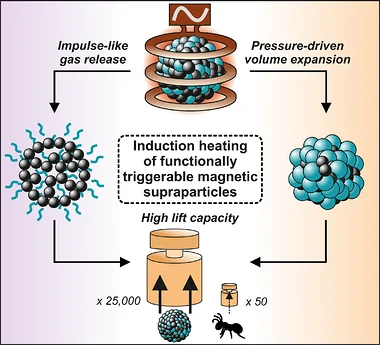

## 1 Introduction

The ability to generate strong and localized mechanical forces at the microscale is central to emerging technologies such as soft microrobotics [1, 2, 3], adaptive materials [4, 5, 6], and responsive interfaces [7, 8, 9]. However, delivering energy to microscopic environments in a controlled and remotely addressable manner remains a fundamental challenge [10, 11, 12]. Many conventional actuation strategies rely on wired electrical inputs, optical access, or global heating, which are difficult to implement in multi‐component or confined systems [13, 14]. Additionally, these approaches often lack control and spatial selectivity [15, 16], with global heating leading to slow activation, thermal gradients, and often collateral material damage [17, 18].

Magnetic nanoparticles (NPs) offer an attractive alternative for remote energy delivery. When exposed to an alternating magnetic field (AMF), they dissipate heat through magnetic losses, enabling contactless and localized induction heating with heating rates of several hundred°C s^−1^ [19, 20, 21]. Because heat generation occurs internally only within magnetically responsive domains, collateral damage of surrounding materials is reduced, and heating can be achieved even in opaque or sealed environments. Owing to the synthetic tunability of parameters such as particle size [22], magnetic anisotropy [23], Curie temperature [24], or heating rate [25], various magnetic fillers have been explored for composite materials (i.e., polymers, scaffolds, ceramics, etc.), with iron oxide NPs such as magnetite (Fe_3_O_4_) [26, 27] and maghemite (γ‐Fe_2_O_3_) [28, 29] playing a prominent role, just as hard magnetic materials like NdFeB [30, 31]. While NdFeB fillers provide strong remanent magnetization and are frequently employed in magnetically actuated systems, iron oxide NPs are among the most widely used magnetic materials owing to their biocompatibility, scalable synthesis, and efficient response to AMFs [32, 33]. More recently, magnetically responsive composites have emerged as promising biomaterials for tissue engineering, where magnetic fillers can provide mechanical reinforcement and allow remote stimulation as well as programmable shape transformations [34]. Recent studies on Fe_3_O_4_‐containing composites and 4D‐printed systems have demonstrated the combination of magnetic responsiveness, improved mechanical performance, and remotely triggered functionality, highlighting the potential for future bone tissue engineering and regenerative medicine applications, as highlighted in recent studies by the Baghani group [35, 36]. In addition to magnetic guidance and actuation, particularly magnetite‐based fillers can serve as localized heat sources enabling remote triggering of self‐healing [37, 38], on‐demand bonding and debonding [39, 40], and shape transformation [41, 42].

In these systems, however, induction heating is primarily used to soften, melt, or thermally degrade the surrounding matrix, thereby reducing interfacial strength. However, systems relying solely on NPs as inductive heat mediators instead of active force‐generating components remain limited, as the resulting heat‐induced mechanical responses are typically confined to the tens‐to‐hundreds‐of‐kPa regime [43]. Consequently, producing substantial mechanical stress or pressure within confined environments remains challenging.

To overcome the limited force generation of purely magnetothermal systems, thermally responsive materials and blowing agents have been investigated as pressure‐generating additives. For example, thermally expandable microspheres have been reported that typically exhibit an up to five‐fold volumetric expansion rate [44, 45, 46], with other studies even showcasing up to 65‐fold increases in volume [47, 48]. Similarly, changes in volume can also be achieved with hydrogel‐ and superabsorber‐based systems when exposed to water, inducing swelling to up to the 300‐fold of the original volume [49, 50]. On another note, agents such as azodicarbonamide (ADCA) have been explored regarding their expansion rate of up to 24% enabled by rapid gas production [51, 52, 53]. These systems have been widely employed in applications for debonding or foaming, where heat‐induced gas formation or volume expansion generates mechanical stress capable of disrupting interfaces or modifying material structures. While these structures can produce substantial expansion rates, they typically rely on global heating procedures or swelling by humidity exposure, resulting in limited spatial and temporal control and often inefficient energy delivery in complex structures.

Comparatively little attention has yet been devoted to systems that directly transform localized heat from induction heating into mechanical work and local pressure through the combination of thermally responsive materials or expanding agents with magnetic NPs. While macroscopic millimeter‐scaled steel‐based capsules able to release a blowing agent upon induction heating have been reported, this work focuses solely on heat‐triggered bursting without quantifying generated stress or pressure [54]. To the best of our knowledge, no study has yet systematically investigated the efficient conversion of electromagnetic energy into mechanical work through pressure‐generating blowing agents directly integrated with inductively heated magnetic NPs in a unified micron‐scale particle platform. Notably, this confinement can substantially amplify this effect: even small amounts of thermally generated gas or phase transitions within restricted volumes may produce considerable internal pressure relative to the system size [55, 56, 57].

A suitable hierarchical design platform to realize this close spatial proximity between magnetic NPs and responsive materials are supraparticles (SPs). SPs are typically synthesized by spray‐drying NP building blocks that are atomized into droplets in a hot chamber and forced into micron‐scale assemblies by solvent evaporation, yielding a free‐flowing powder [58, 59]. This modular architecture enables high functional loading, tunable composition, and controlled spatial organization when appropriately integrated as an additive. As a result, SPs provide a versatile route to bridge nanoscale heat generation with microscale mechanical actuation.

To address this gap between heat generation and mechanical actuation, two inductively triggerable SP systems are synthesized via spray‐drying magnetic NPs together with blowing agents that exhibit distinct expansion mechanisms: one relies on thermally induced decomposition leading to rapid gas release, while the other is governed by water evaporation within the SP structure, resulting in volumetric expansion. Their heat generation, compositional tunability, micron‐scale morphology before and after induction heating, and macroscopic expansion behavior are systematically characterized and compared to external thermal activation, with elucidation of the respective working mechanisms.

Both systems are subsequently incorporated into an epoxy matrix to investigate force generation in application‐relevant material systems. The ability to produce localized mechanical stresses through magnetically triggered expansion provides an attractive strategy for controlled material separation and interface disruption. In bonded or composite structures, such localized expansion could generate stresses sufficient to overcome adhesive or cohesive interactions, enabling targeted debonding, disassembly of multi‐material structures, or selective release processes [39, 60]. These concepts of interactive materials are particularly relevant for opening thermoset‐based materials, which provide excellent mechanical performance but remain permanently cross‐linked and therefore challenging to circular material strategies [61, 62, 63, 64].

Magnetically driven force generation offers new opportunities for remotely powered microscale actuators and switches. Architectures that integrate induction heating with hierarchical material design may enable programmable mechanical responses that can be triggered without direct contact or wiring. Such concepts could support the development of adaptive material systems for remotely powered micron‐scaled actuation and advances material concepts toward responsive and sustainable structural systems.

## 2 Results and Discussion

### 2.1 Design Concept of Inductively Triggerable Functional Supraparticles

Inductively triggerable SPs are synthesized by spray‐drying two main building blocks: magnetic NPs to enable localized induction heating (Figure 1a) and blowing agents to generate functionality upon the inductive trigger (Figure 1b). As for magnetic NPs, tuning and choosing the right properties for the envisioned functionality and application is of major importance, especially regarding the induction heating rate that determines how fast the temperature rise occurs, and the maximum heating temperature that can be regulated by a set material‐inherent Curie temperature. For this, two different magnetic NP types are chosen, namely ferrimagnetic iron oxide nanoparticles (FIONs, Fe_3_O_4_) (Figure 1a1) and ZnCo ferrite (ZnCoFe_2_O_4_) NPs (Figure 1a2). Octahedral FIONs were synthesized by an oxidative precipitation method and subsequently functionalized with citric acid to ensure better stability in aqueous dispersion [65]. These NPs exhibit a high initial heating rate of approximately 1000 K s^−1^ with maximum reached temperatures of 570°C during induction heating (see Figures S1a1, S1c). This stems from their large mean size around 50 nm, a saturation magnetization of 90 emu g^−1^ and a coercivity of 50 Oe (see Figures S1b, S1d), ideal for maximum heat generation at the herein chosen induction heating conditions with an AMF amplitude of 50 mT and a frequency of 1.4 MHz. Spherical ZnCo ferrite NPs were obtained from a spray‐drying based approach with a subsequent annealing step [24], displaying a slightly lower initial heating rate of 800 K s^−1^ and, as the most significant difference, a lower Curie temperature at 220°C (see Figures S1a2, S1c). These NPs are smaller in size (around 20 nm) with a similar magnetic moment around 80 emu g^−1^, but a coercivity of only 5 Oe, that is, one order of magnitude lower than for FIONs (see Figures S1b, S1d). Different NPs were chosen with regard to future applications of the triggerable systems; while fast and high‐temperature heating of NPs is most efficient and often leads to the biggest effects, the maximum temperature has to be controlled in many cases so as not to damage the surroundings or overheat in applications with thermal limitations.

**FIGURE 1:**
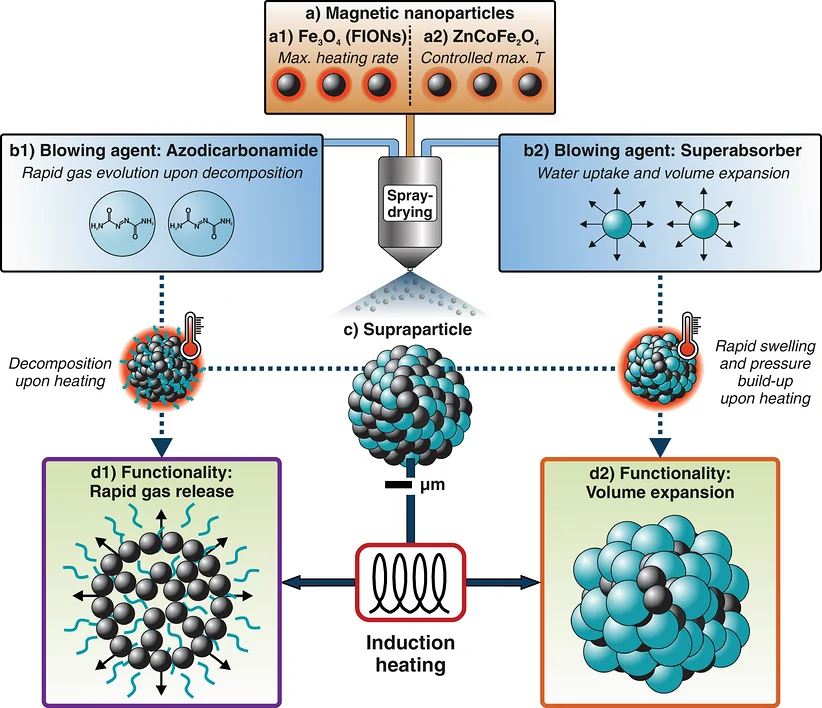
Schematic concept of inductively triggerable SPs with different functionalities. Spray‐drying magnetic NPs (a), either FIONs with maximum heating rates (a1) or ZnCoFe_2_O_4_ NPs with a controlled maximum temperature (a2), together with blowing agents (b), either azodicarbonamide (ADCA) with rapid gas evolution upon decomposition (b1) or superabsorbers that can take up water and expand in volume (b2), yields functional micron‐scale SPs (c). Upon induction heating, these SPs exhibit different functional principles: rapid gas development for ADCA‐based structures (d1) or strong volume expansion for superabsorber‐based systems (d2).

In addition to magnetic NPs, two distinct blowing agents were selected that inherently lead to different expansion mechanisms. On the one hand, ADCA is a well‐known chemical blowing agent, an azo compound (SEM see Figure S2a1) which rapidly generates gas upon thermal decomposition [66, 67]. Correspondingly, thermogravimetric analysis (TGA) data indicates a substantial mass loss around 200°C (see Figure S2b). On the other hand, poly(acrylamide‐*co*‐acrylic acid) (P(AAm‐*co*‐AAc)) is a superabsorbent polymer (SEM see Figure 2a2) that can take up large amounts of water with swelling degrees of more than 200% [68]. When this water is evaporated again upon heating to around 100°C, volume expansion sets in, while the polymer itself remains relatively stable up to temperatures of 250°C, after which decomposition processes begin according to TGA (see Figure S2b). As both blowing agents rely on initial gas evolution to exhibit their respective functionality, the local temperature is also limited by the generated gas, thus preventing overheating and posing as a built‐in safety feature.

**FIGURE 2:**
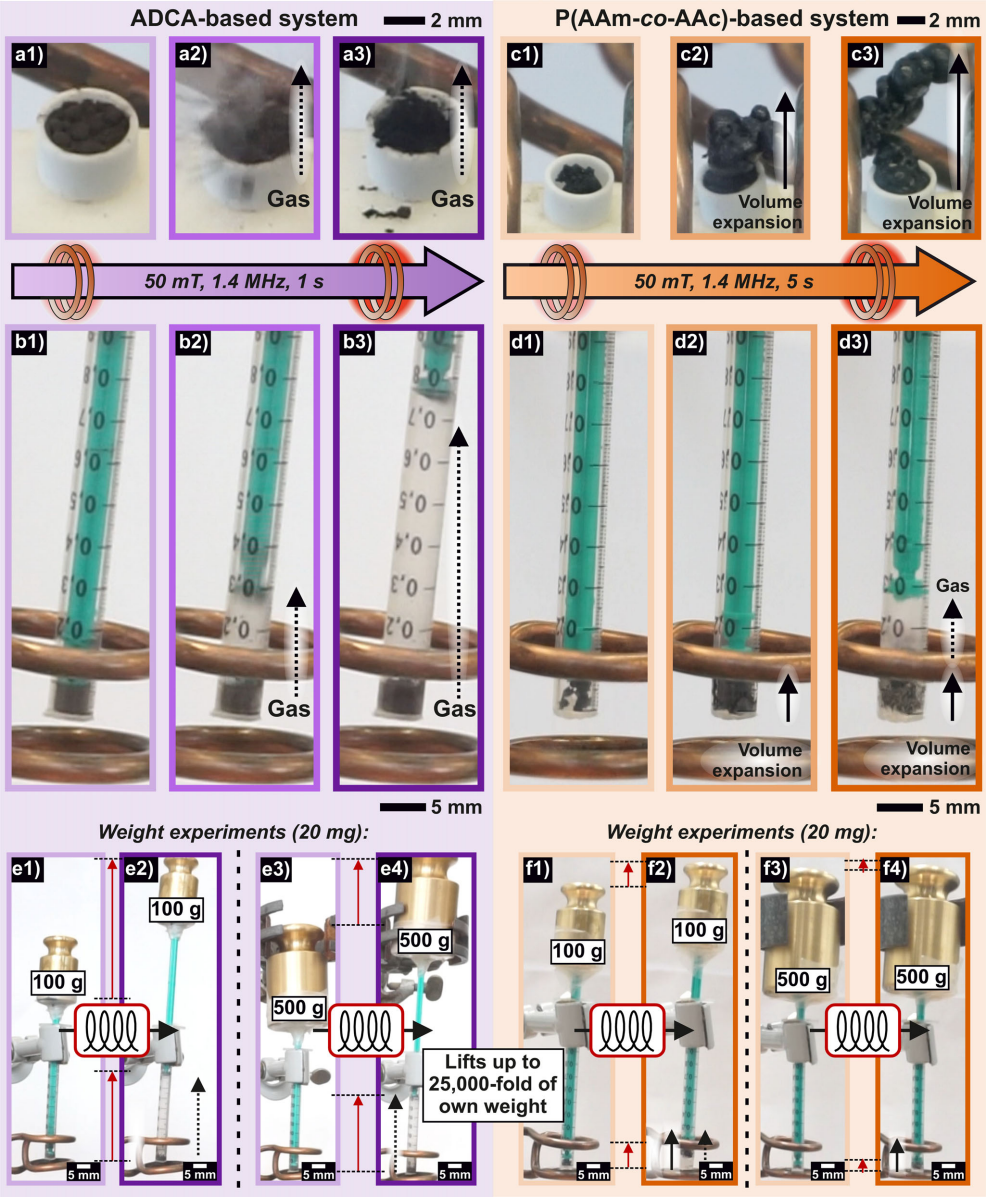
Macroscopic gas generation and volume expansion effects by inductively triggerable SP systems. (a) Inductively triggering ADCA:FIONs 1:1 SPs in a crucible results in a burst, with large amounts of gas produced in below 1 s as showcased in (b). (c) Inductively triggering pre‐treated P(AAm‐co‐AAc):FIONs 8:2 SPs in a crucible results in substantial volume expansion within 5 s, with less gas formation as shown in (d). e1‐2) and f1‐2) display that 20 mg of SP material can lift 100 g, with e3‐4) and f3‐4) showing that even 500 g can be lifted even with existing friction, equivalent to the 25 000‐fold of the utilized sample mass.

For both blowing agents, respective SPs were created by spray‐drying them together with magnetic NPs (Figure 1c). The SP architecture allows an intimate spatial placement of the localized internal heating source, that is, magnetic NPs, and the functional component, that is, the blowing agent. This is hard to achieve otherwise yet crucial to allow for an instantaneous trigger of the envisioned functionality, all within one SP entity. The functionality itself depends on the utilized blowing agent: in ADCA‐based SPs, decomposition of ADCA sets in when it is exposed to the heat produced by the magnetic NPs, leading to a rapid evolution of large amounts of gas from the SP reservoir, connected with pressure development (Figure 1d1). In SPs based on P(AAm‐*co*‐AAc), the structure swells through water uptake when subjected to humidity, before induction heating leads to water evaporation and subsequent volume expansion by orders of magnitude through internal pressure build‐up (Figure 1d2). Hence, two distinct SP functionalities are displayed, both based on initial gas production: rapid decomposition of ADCA resulting in instantaneous gas release on the one side, and internal gas vapor build‐up leading to substantial volume expansion by using a superabsorber on the other side.

### 2.2 Investigation of Inductively Triggerable Macroscopic Supraparticle Functionalities

Both functionalities are first demonstrated on the macroscopic scale during induction heating experiments (50 mT, 1.4 MHz), tested each in an Al_2_O_3_ crucible and a syringe setup with a flat bottom to monitor both gas formation and volume expansion separately in each system. In the investigated model systems, FIONs are employed to achieve maximum induction heating power and the maximum achievable effect on short time scales. For the ADCA‐based model system (ratio ADCA:FIONs 1:1), an instantaneous burst occurs in the crucible after induction heating for as short as 1 s, corresponding to a rapid gas formation by the decomposition of ADCA (Figure 2a1–a3, corresponding video is available online, Supplementary Video S1). The blurriness of the snapshot (2a2) confirms the rapidness of the burst, which cannot be sharply resolved by the used camera system. As ADCA makes up 50% of the entire SP mass in this system and almost completely transforms into gaseous products upon decomposition (∼ 220 mL g^−1^) [69], the effect is instantaneously seen also on the macroscopic scale. This is further underlined when considering the syringe setup where gas is produced already in less than 1 s after the induction trigger from only 20 mg of SP powder, showcasing the sheer pressure and gas amounts generated by this approach (Figure 2b1–b3, corresponding video is available online, Supplementary Video S2).

The P(AAm‐*co*‐AAc)‐based model system (ratio P(AAm‐*co*‐AAc):FIONs 8:2) is subjected to intermediate treatment steps prior to induction heating, consisting of exposing the SPs to humidity to allow water uptake into the structure, followed by oven drying at 100°C to remove loosely adsorbed excess water. A lower amount of FIONs is chosen in this model system as the heating temperature should be high enough to evaporate water out of the structure, but low enough to prevent polymer decomposition, which are factors that do not have to be considered in the ADCA‐based case. Therefore, a slightly longer induction heating pulse is chosen to allow sufficient heat production. Within 5 s, a substantial volume expansion is seen in the crucible, where the volume of 30 mg of pre‐treated substance grows by a multitude upon the trigger (Figure 2c1–c3, corresponding video is available online, Supplementary Video S3). As opposed to the simple gas generation that the ADCA‐based samples are characterized by, this system is dominated by the extensive volume expansion and only minor additional gas production in the setup, as most of the produced gas stays inside of the structure and drives the expansion (Figure 2d1–d3, corresponding video is available online, Supplementary Video S4).

For both systems, notably, the functional performance is retained even after prolonged air exposure (with UV light and changing temperatures) for one year, as evidenced by unchanged gas generation and volume expansion (see Figure S3). This long‐term ambient stability is non‐trivial, as gas‐generating and volume‐responsive systems are typically susceptible to deactivation through moisture uptake, oxidation, or loss of volatile components [70, 71, 72, 73]. In contrast, the active components in the presented systems appear suitably protected from environmental deactivation. While functional stability does not preclude chemical or structural ageing, this shows that such changes do not measurably affect the macroscopic performance, which can be considered a notable benefit.

The macroscopic tests underline the fundamentally different working principles of both systems that can be achieved simply by exchanging the blowing agent during spray‐drying. Independent of the working mechanism, both presented systems display enormous forces when different weights are placed on top of syringes containing 20 mg of (if necessary pre‐treated) SP material. For both systems, not only weights of 100 g but also remarkably of 500 g could easily be lifted within mere seconds, displaying a lifting capability of the 25 000‐fold of the employed material weight (Figures 2e1–4 and 2f1–4; corresponding videos are available online, Supplementary Videos S5–S8).

**FIGURE 3:**
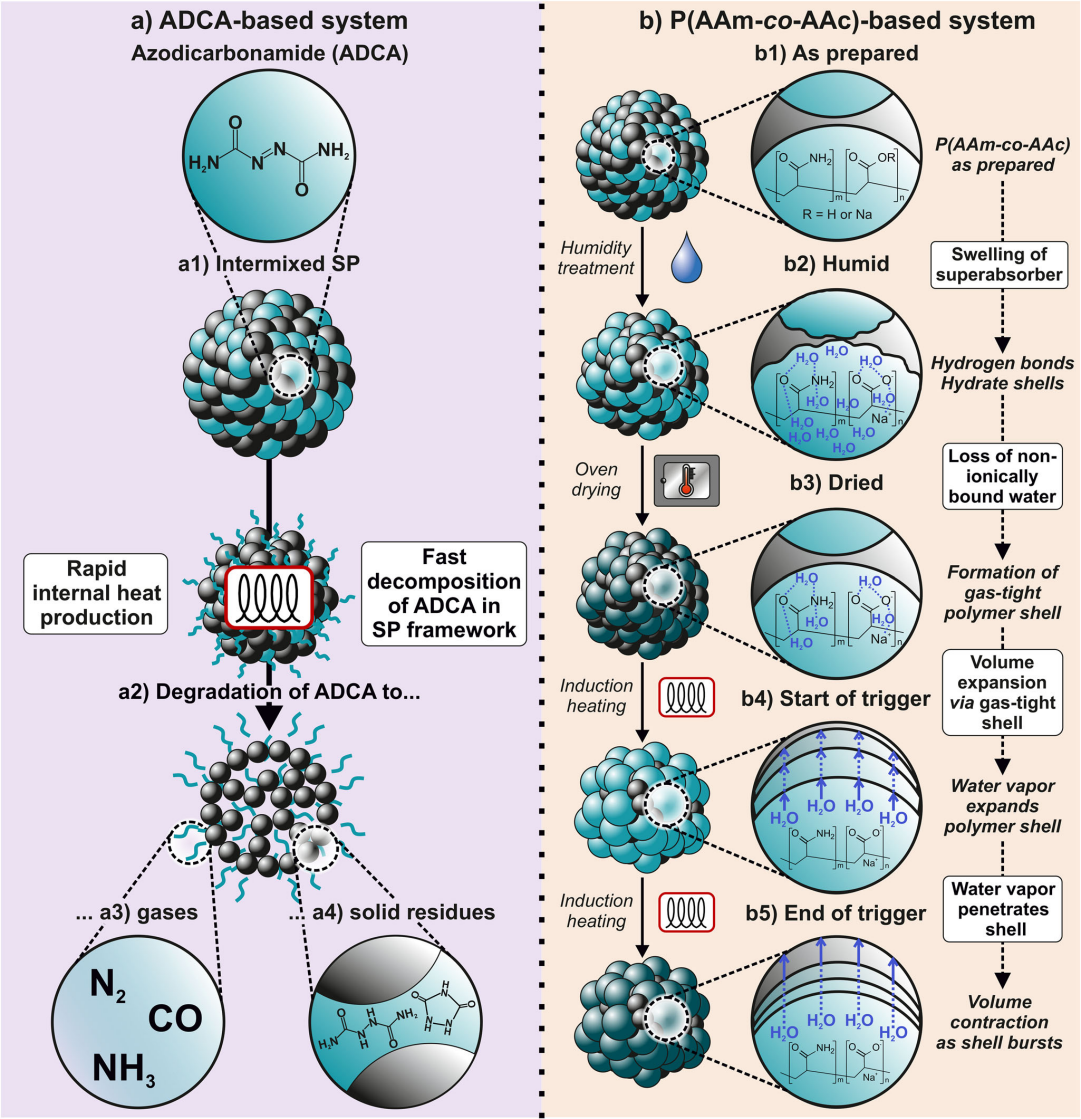
Mechanism for gas generation and volume expansion from inductively triggered SPs. (a) ADCA‐based system: intermixed SPs (a1) are inductively heated, causing the fast decomposition of ADCA (a2) to mostly gases (a3) and solid residues (a4). (b) P(AAm‐co‐AAc)‐based system: intermixed SPs (a1) are pre‐treated with humidity (a2), causing the swelling of the superabsorber, before non‐ionically bound water is lost by oven drying to go into the dried state (a3) and form a gas‐tight polymer shell. Induction heating causes water vapor to expand the polymer shell (b4), before the shell bursts and volume contracts at prolonged heating (b5).

**FIGURE 4:**
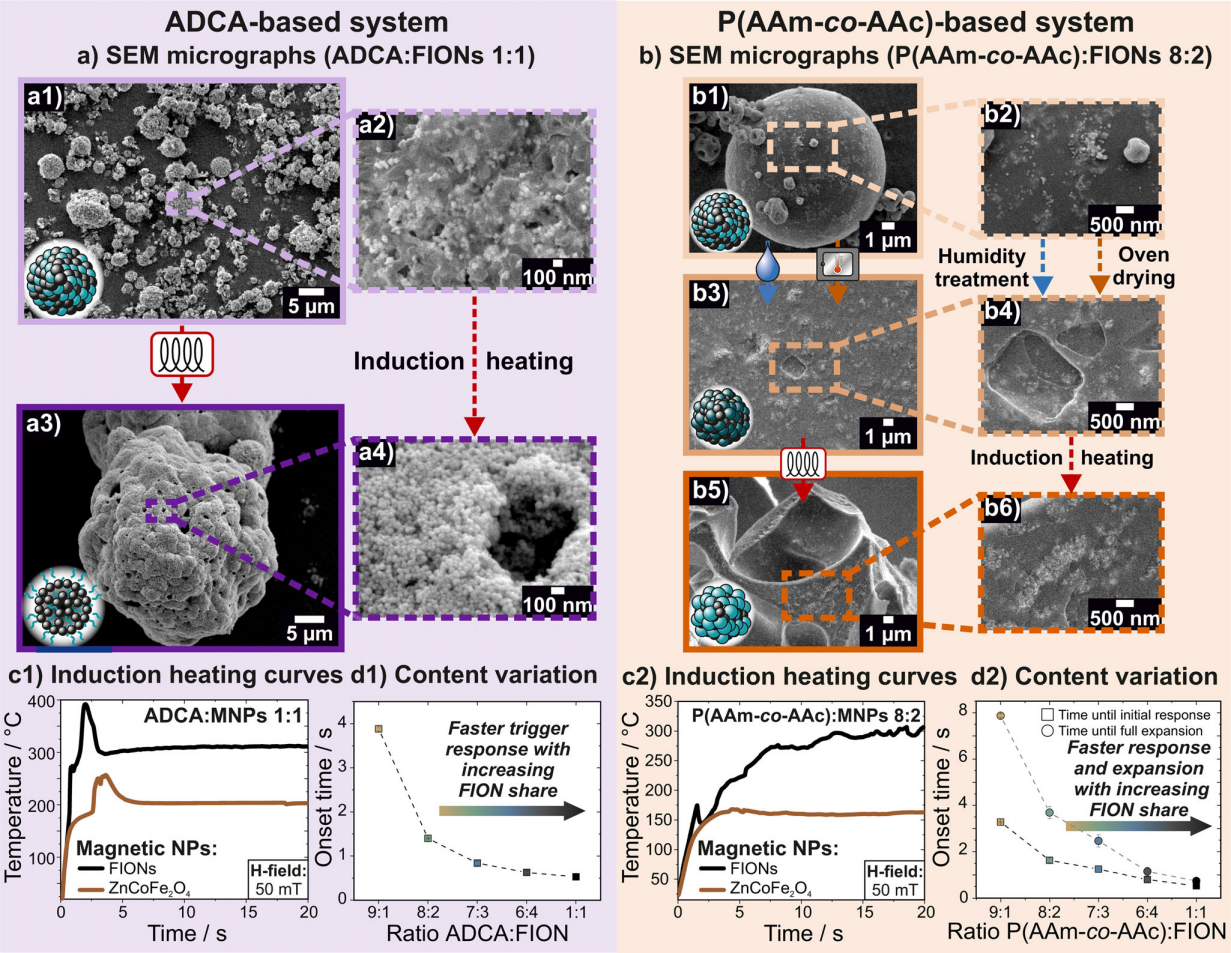
Characterization of inductively triggerable SP systems. (a) SEM micrographs of the ADCA‐based system before (a1‐2) and after (a3‐4) induction heating, showing the formation of multi‐micron FION networks with macropores upon the trigger. (b) SEM micrographs of the P(AAm‐*co*‐AAc)‐based system after spray‐drying (b1‐2), after humidity treatment and drying (b3‐4), and after induction heating (b5‐6). Humidity treatment leads to swelling of the superabsorber, while evaporating water in induction heating results in pore formation. (c) Variation of the inductively heatable NP component in the ADCA‐ (c1) and P(AAm‐*co*‐AAc)‐based system (c2), with ZnCo ferrites introducing lower maximum temperatures. (d) Variation of the ratio of blowing agent to FION in the ADCA‐ (c1) and P(AAm‐*co*‐AAc)‐based systems (c2), with increasing FION share resulting in a faster trigger response. Onset times were determined as the average of three individually read out times during heating in a crucible.

While the resulting calculated energy conversion during these lifting experiments is low (< 0.01%) (see Table 1), the objective of this system is not efficiency but rather the generation of sufficiently fast and localized actuation to trigger material expansion. When considering total values of energy consumption, however, these add up to merely 2.8 Wh for the P(AAm‐*co*‐AAc)‐ and 0.7 Wh for the ADCA‐based system that differ in the required induction heating times to achieve expansion. In this regard, it is notable that for the ADCA‐based system, more gas is produced in the syringe when lifting 100 g compared to 500 g, as higher weight increases the counter pressure. Hence, the gas has to do more mechanical work to lift the weight, therefore finding itself at higher pressure and reduced visible volume according to Boyle's law [74]. Accordingly, the work output is increased from 59 ± 18 to 102 ± 46 mJ (relating to 2.9 ± 0.90 and 5.1 ± 2.3 J g^−1^, respectively) when increasing the weight from 100 to 500 g, with a correspondingly increased power density from 2.9 ± 0.9 to 5.1 ± 2.3 W g^−1^ (Table 1, for exact equations regarding the determination of the quantities, see Supporting Information).

**TABLE 1:** Calculation of pressure, syringe plunger displacement, work output, energy conversion efficiency, and gravimetric power density for the lifting experiments with 100 and 500 g, respectively, for both the ADCA‐ and P(AAm‐*co*‐AAc)‐based inductively expandable SP systems. The data was obtained as an average of five individual lifting experiments each. The actuation performance was maintained through all repetitions, with zero observed failures. Consider that the total energy consumption of the ADCA‐based system is 0.7 Wh for a heating time of 1 s and that of the P(AAm‐*co*‐AAc)‐based system 2.8 Wh for a heating time of 5 s. For every experiment, 20 mg of SPs were used.

System	Weight/g	Pressure/bar	Displacement/mL	Work output/mJ	Work per mass/J g^−1^	Energy conversion efficiency/%	Power density/W g^−1^
ADCA	100	0.78	0.8 ± 0.2	59 ± 18	2.9 ± 0.9	2.4·10^−3^ ± 0.8·10^−3^	2.9 ± 0.9
ADCA	500	2.77	0.37 ± 0.15	102 ± 46	5.1 ± 2.3	4.2·10^−3^ ± 1.9·10^−3^	5.1 ± 2.3
P(AAm‐*co*‐AAc)	100	0.78	0.19 ± 0.11	15 ± 8	0.7 ± 0.4	0.15·10^−3^ ± 0.08·10^−3^	0.150 ± 0.08
P(AAm‐*co*‐AAc)	500	2.77	0.21 ± 0.15	59 ± 43	3.0 ± 2.1	0.6·10^−3^ ± 0.4·10^−3^	0.6 ± 0.4

In the P(AAm‐*co*‐AAc)‐based system, gas formation is seen in addition to volume expanse when lifting 100 g as opposed to 500 g. This is due to the generated gas having to work against the viscosity of the simultaneously expanding polymer surrounding it. It is important to mention here that a higher load therefore does not mean that less gas is produced, but rather that gas is stored at higher pressure or redirected into volume expansion. Due to the different working mechanism, that is, pressure generation through macroscopic increase in volume, the maximum reached values of work output (59 ± 43 mJ) and power density (0.6 ± 0.4 W g^−1^) are lower than in the ADCA‐based system, as the values are calculated from the original syringe displacement, which is lower with less pronounced gas formation (Table 1). However, the remarkable power per mass with a work output of up to 3 J g^−1^ generated from a system working dominantly via the expansion of its own volume should still be underlined, in a principle similar to a hydraulic jack.

The comparatively high lifting capacities and power density values highlight the exceptional performance of the displayed SP systems in converting their own mass into mechanical work. Reported power densities exceed literature values for biological skeleton muscles (typically 50.000 W g^−1^) [75], artificial muscles (50.000–60.000 W g^−1^) [76, 77], and even biomimetic soft actuators (exceeding 100.000 W g^−1^) [78], with figures close to those of advanced thermal actuators (more than 5.000 kW g^−1^) [79]. Regarding lifting capacities, even advanced artificial actuators and robots reach values of only 10^2^ to 10^4^ their own weight, which is considered extraordinary in both materials and robotics context (e.g., MoS_2_ actuators [80], soft robots [81, 82], or carbon‐fiber artificial muscles [83]). Herein, we report systems capable of lifting 25 000 times their own mass (assuming that friction is negligible), which surpass most literature‐known high‐performance artificial actuators by more than one order of magnitude. This places the systems in rare regimes of mechanical functionality, challenging typical scaling expectations due to a combination of specific structural mechanisms and novel microstructures. The presented systems are comparable only to extreme biological outliers that are used as biomimetic inspiration for high‐load, lightweight systems [84].

### 2.3 Mechanism of the Inductively Triggered Material Expansion

Considering the enormous macroscopic effect of both systems, the underlying nano‐ and micron‐scale mechanism has to be investigated to elucidate how gas generation and volume expansion in the SP materials happen in detail. Herein, the mechanism is fundamentally different for both systems employing different blowing agents. In the ADCA‐based system, the working principle is dominated by the well‐known chemical decomposition of ADCA (Figures 3a, and S4) [85, 86]. In pristine SPs, ADCA is intact and intermixed with magnetic NPs (Figure 3a1). Upon rapid localized heat production by magnetic NPs in an AMF, the thermally induced decomposition of neighboring ADCA is instantaneously erupted within one second (Figure 3a2). Consequently, this degradation primarily leads to the formation of gases, foremost N_2_ (65%–72%) but also CO (24%–26%), CO_2_ (1%–5%), and NH_3_ (0.5%–5%), explaining the visible production of gases even after 1 s of induction heating (Figure 3a3) [69, 87]. Note that although NH_3_ and CO are produced as side products, the quantities generated in the present laboratory‐scale system are low and are not expected to pose significant safety concerns under standard ventilation conditions. For future large‐scale or embedded applications, appropriate ventilation and gas management strategies should be considered.

Noteworthily, the gas release is a burst rather than a slow gas diffusion. This is due to the almost instantaneous heat transfer of NPs to ADCA, which are in direct contact. Thereby, heat transportation delays from the heat source to the blowing agent are negligible. In addition, solid side products in the form of urazol and biurea are formed and remain in the SP structure instead of evaporating (Figure 3a4). The latter are confirmed by TGA, as mass loss is still detectable in samples that were inductively triggered, meaning that ADCA is not fully decomposed to gaseous products under the presented conditions (see Figure S5). Additionally, attenuated total reflection infrared (ATR‐IR) and magnetic particle spectroscopy (MPS) investigations confirm residual ADCA components as well (discussion and data shown in Supporting Information, Figures S6a and S7a).

The mechanism of the P(AAm‐*co*‐AAc)‐based system, in contrast, is dominated by volume expansion through water vaporization from the humidity‐treated superabsorber (Figure 3b). In pristine spray‐dried SPs, magnetic NPs are intermixed with the superabsorbent polymer, in this case P(AAm‐*co*‐AAc) as partial sodium salt (M_W_ = 520 kg mol^−1^, acrylamide ∼ 80 wt%) (Figure 3b1). However, no expansion is possible in this state, as water uptake into the superabsorber structure is needed for a later trigger. This is achieved by humidity treatment, leading to swelling of P(AAm‐*co*‐AAc) (Figure 3b2). In doing so, water is bound to the polymer structure in several forms, primarily as hydrate shells and hydrogen bonds at ionic or polar groups, literature‐known as strongly primary bound “non‐freezing” water [88]. In addition, secondary bound water is loosely coordinated near hydrated ionic domains to already bonded water, referred to as clustered “freezable bound” water. Remaining free water is further apparent in nano‐ or micropores as capillary quasi‐bulk water domains [88]. This is confirmed by ATR‐IR and MPS measurements (see Figures S6b and S7b).

After water uptake, a subsequent drying step is performed in the oven to lose all secondary bound and free water, so that only strongly, primarily bound water remains in the structure (Figure 3b3). While water evaporates, polymer chains on the surface collapse and the network contracts, leading to a dense skin layer with low free volume, decreased mobility, and thus reduced gas permeability [89, 90]. Due to this, the matrix now behaves like a dry glassy polymer in which primary bound water exists as isolated hydration shells and does not percolate due to low diffusivity [91]. With this process, a semi‐stable state is created that will not change under ambient conditions.

After this necessary pre‐treatment, the induction heating trigger can set in, leading the magnetic NPs to produce heat on a fast timescale within the gas‐tight polymer matrix, which in turn leads to evaporation of primary bound water. During the evaporation process, the emerging internal water vapor meets the shell that expands through the sudden pressure, while the gas is not yet exiting the structure (Figure 3b4). As the polymer relaxation time is significantly slower than the induction heating time (s to min vs. µs to ms), the gaseous transformation of water inside the superabsorber happens quickly, while the diffusion of the vapor through the polymer is slow [92, 93]. Hence, the internal vapor pressure exceeds the local mechanical strength of the swollen polymer, causing extensive volume growth in response to induction heating. Additionally, gas formation induces a structural rearrangement during the evaporation, again causing magnetic NPs to be more spatially separated. The resulting changed MPS signal further confirms the new mechanical state of the polymer environment and irreversible matrix reorganization (see Figure S7b). At a certain point, water vapor can penetrate the shell, due to the increasing internal pressure and the rising induction heating temperature, as viscosity is temperature‐dependent (Figure 3b5) [94]. In turn, this results in volume contraction of the material as the superabsorber shell bursts. At this point, decomposition is indicated in the ATR‐IR due to the decrease or disappearance of characteristic P(AAm‐*co*‐AAc) vibrations (see Figure S6b). Therefore, for maximum effect, induction heating has to stop either before the polymer decomposes or by using Curie temperature‐controlled magnetic components.

### 2.4 Characterization and Variation of Inductively Triggerable Supraparticle Systems

A thorough characterization of both mechanistically different inductively triggerable systems is conducted on the nano‐ and micron‐scale to further confirm the proposed mechanisms and understand how individual SPs behave, especially by considering morphological changes after induction heating compared to the pristine state in scanning electron microscopy (SEM). For the ADCA‐based system, spray‐drying occurred in dimethyl sulfoxide (DMSO) as opposed to water, as ADCA dissolves well in DMSO but precipitates in contact with water [95]. Precipitation leads to large ADCA components within the center of spray‐dried SPs, yielding an uneven distribution with magnetic NPs. This is observed for spray‐drying both components in water as well as for spray‐drying an aqueous dispersion of magnetic NPs with ADCA dissolved in DMSO (see Figure S8). In contrast, spray‐drying both ADCA and magnetic NPs in DMSO makes for a more uniform distribution of both components in quasi‐spherical SPs on the lower micron‐size range, as seen for the chosen model system with a 1:1 ratio of ADCA to FIONs (Figure 4a1). We hypothesize that structure control in this regard is important for uniform heat distribution during induction heating. Herein, FIONs are well intermixed with ADCA, with the surface of SPs showing small interstitial pores in an otherwise even surface (Figure 4a2). However, after induction heating, no individual SPs are distinguishable anymore. Instead, new multi‐micron structures based on a FION framework with large macropores are formed, in which no ADCA is visible due to its heat‐induced decomposition (Figure 4a3‐4). The formation of enlarged restructured FION networks is presumably favored by pressure build‐up on the millisecond timescale with no gas diffusion path out, which in the end results in internal overpressure blowing the SP structure apart. This thermal shock then effectively produces macropores by cracking, as the gas pressure from decomposing ADCA is locally increased due to typical gas yields between 250 and 350 mL per gram [67]. During decomposition of ADCA, the mobility of the NPs distributed within the ADCA matrix increases, while they are pushed apart by the expanding gas but remain connected so that neighboring SP can merge. The resulting interconnected magnetic networks are then presumably held together by solid side products of ADCA decomposition.

The P(AAm‐*co*‐AAc)‐based system is spray‐dried with FIONs in a ratio of 8:2 to ensure sufficient polymer swelling upon humidity exposure and moderate induction heating temperatures. Initial spray‐drying leads to spherical SPs with FIONs uniformly embedded in the superabsorber matrix that is spread over the entire SP (Figure 4b1‐2). Humidity treatment and subsequent oven drying results in loss of the SP structure, as P(AAm‐*co*‐AAc) swells by a multitude and now resembles a bulk material with FIONs uniformly distributed (Figure 4b3‐4). Humidity treatment was performed at 98% relative humidity for 4 h. Lower humidity levels have been tested, with the initial SP structure still apparent in the SEM up to levels of 80% (see Figure S9a). Generally, the water uptake increases exponentially when increasing the relative humidity in 10% steps, with 98% relative humidity corresponding to the maximum possible uptake within 4 h, that is, 44% of the material mass. Exposure time was also varied between 1 and 24 h, with the water uptake increasing significantly up to 8 h and flattening at 24 h (see Figure S9b). An exposure time of 4 h was chosen as a trade‐off between time constraints and manageability, as higher water uptake at longer exposure times makes the sample behave more like a viscous liquid, complicating handling.

After induction heating, the overall structure of the pre‐treated state is maintained, however, large macropores are visible in the material with an increased surface roughness (Figure 4b5‐6). Through the evaporation of water from the superabsorber structure and the pressure build‐up against the gas‐tight polymer matrix by the extensive volume expansion, pores are formed at the position where accumulated vapor left the structure, leading to mechanical failure of the polymer network. Caused by the rapid evaporation kinetics, these pores grow catastrophically rather than uniformly, hence the large uncontrolled sizes.

In model SPs for both functionalities, FION heating leads to immediate effects due to the high temperatures achieved during induction heating and the corresponding high heating rates. However, depending on a final application, this is not always favorable, as surrounding materials could take damage. To avoid excessive heating, magnetic NPs with high heating rates and lower Curie temperatures can be employed, such as ZnCo ferrites (see again, Figure S1). For both systems, this change in heatable component lowers the maximum physically achievable induction heating temperature from 300°C to 200°C or 160°C for the ADCA‐ and P(AAm‐*co*‐AAc)‐based systems, respectively (Figure 4c), while the successful trigger is confirmed by structures comparable to FION‐based systems in the SEM (see Figures S10 and S11). In both cases, a slower initial response to the induction trigger is noted when employing ZnCo ferrites compared to FIONs, due to the slightly diminished heating rate. Consequently, achieving a more controlled surrounding temperature goes along with minor delays in the response time (see Figures S10 and S11).

The major difference and benefit, however, lies in the P(AAm‐*co*‐AAc)‐based system, as achieving a lower temperature with ZnCo ferrites prevents polymer decomposition, evidenced by the more regular induction heating curve compared to visible decomposition‐induced gas formation when FIONs are used (see Figure 4c2). This effect now allows a certain reversibility that is inherent to the P(AAm‐*co*‐AAc)‐based system, as ADCA decomposes irreversibly. Reversibility was tested by exposing the samples to humidity again after initial induction heating and subsequent induction heating (see Figure S12). Again, successful expansion was achieved after inductively heating for 30 s, demonstrating the reversibility by avoiding polymer decomposition. In comparison, in the FION system, reversibility can only be obtained when controlling the induction heating time at a maximum 5 s during the first induction heating run. At longer heating exposure, the polymer starts decomposing, preventing re‐activation by humidity and thus a trigger during the second induction heating run.

While features and functionalities are shown for the displayed model ratios of both systems, ratio variations are easily feasible and quickly adaptable owing to the underlying spray‐drying procedure, which allows ratio variation by mixing the used building blocks in respective ratios before spray‐drying. Consequently, blowing agent:FION ratios between 1:1 and 9:1 were tested. The corresponding ratios are confirmed by TGA, where the mass loss corresponds to the amount of blowing agent lost, and superconducting quantum interference device (SQUID) measurements, where the mass‐normalized magnetic moment is lower for a diminished FION share (see Figure S13), in agreement with expectations. In both systems, the trigger response is generally faster with increasing FION share (Figure 4d; for exact onset times in both systems see Table S2). However, naturally this comes at the cost of a lower effect due to a lower amount of the blowing agent that is responsible for the functionality. In this regard, for the P(AAm‐*co*‐AAc)‐based system, with increasing FION share, not only the initial response to the trigger is accelerated, but also the time until full volume expansion (e.g., 7.5 s for 9:1 and 1 s for 1:1). The faster response is further evidenced in the corresponding induction heating curves with significantly increased initial heating rate and temperatures reached for higher FION content (see Figure S14). Nevertheless, these also display the successful trigger that is temporally delayed with higher blowing agent content, as indicated by the sudden sharp temperature decrease in the ADCA‐ and the gradual thermal increase after the short plateau in the P(AAm‐*co*‐AAc)‐based system.

Calculations for the mass‐normalized work output of the ADCA‐based system are in accordance with expectations, displaying an increase with increasing blowing agent content (see Table S1). This is due to a higher amount of produced gases with a higher ADCA share, contributing to a larger volume displacement in the syringe used for the experiments. However, for the P(AAm‐*co*‐AAc)‐based system, it is important not only to consider the overall work output, as this seems to decrease with a larger blowing agent share, which is due to the additional decomposition of the superabsorbent polymer (see also Table S1). In contrast, when contemplating only the material expansion itself (which is the prominent working mechanism), it becomes obvious that this volume change increases with increasing amount of P(AAm‐*co*‐AAc), signifying a larger lifting capability by the material force itself. The same trend is seen when normalizing the volume change to the blowing agent mass, however not when normalizing to the magnetic NP mass, supporting the assumption that this is purely due to the increase in blowing agent (see Table S3). From a practical point of view, this means that the amount of blowing agent has to be chosen depending on the desired force needed for an application, while the remaining amount of magnetic NPs determines the trigger time and energy consumption. In this regard, a higher share of blowing agent will signify a larger power density but prolonged energy consumption and onset times. Independent of the ratio and system, no failures were observed, and reproducibility was maintained over three individually performed experiments for each ratio and system, respectively.

Overall, for both cases, the careful tuning of the ratio of blowing agent to magnetic NPs is of crucial importance to achieve the desired functionalities with sufficient temperature for the trigger, while avoiding overheating the material system. Additionally, choosing the desired blowing agent‐to‐magnetic NP ratio is important when evaluating whether a fast trigger or the maximum mechanical output is needed, in the form of work in the ADCA‐based system or in the form of the volume change of the material itself in the P(AAm‐*co*‐AAc)‐based system. A more detailed discussion about the ratio variation is included in the Supporting Information (see also Figure S15 and Figure S16). Additionally, with mechanism and morphologies understood, the P(AAm‐*co*‐AAc)‐based system in particular can be optimized regarding its volume expansion capability by modifying the SP contents or structure. In this regard, either adding NaHCO_3_ as an additional gas‐generating component or changing the SP structure itself from intermixed to core‐shell proves useful design parameters to increase volume expansion and tune response times. This is more thoroughly discussed in the Supporting Information (see also Figures S17 and S18).

### 2.5 Comparison of Global Oven Heating and Local Induction Heating

Inherent to the proposed mechanism is the use of induction heating instead of conventional external heating methods. In global heating (e.g., an oven), heat is transferred from the surroundings to the material, resulting in temperature gradients between the sample surface and core (Figure 5a). In contrast, induction heating generates heat directly within the material via AMF exposure, leading to a more uniform internal temperature distribution (Figure 5b) [21]. An inductively heated SP therefore exhibits both a hot core and surface, as it acts as the heat source itself. Outside the material, heat dissipates rapidly, creating comparatively cold surroundings which helps prevent overheating or damage to adjacent components [96, 97]. Importantly, when using induction heating, the surrounding matrix will always be equally hot or colder than the heating SPs. In contrast, upon global heating, heat diffuses through the matrix to the SPs so that the matrix is always hotter or equally hot compared to the SPs. Based on these physically different concepts, heat exposure of the matrix—which is typically undesired—is reduced by employing induction heating compared to global heating.

**FIGURE 5:**
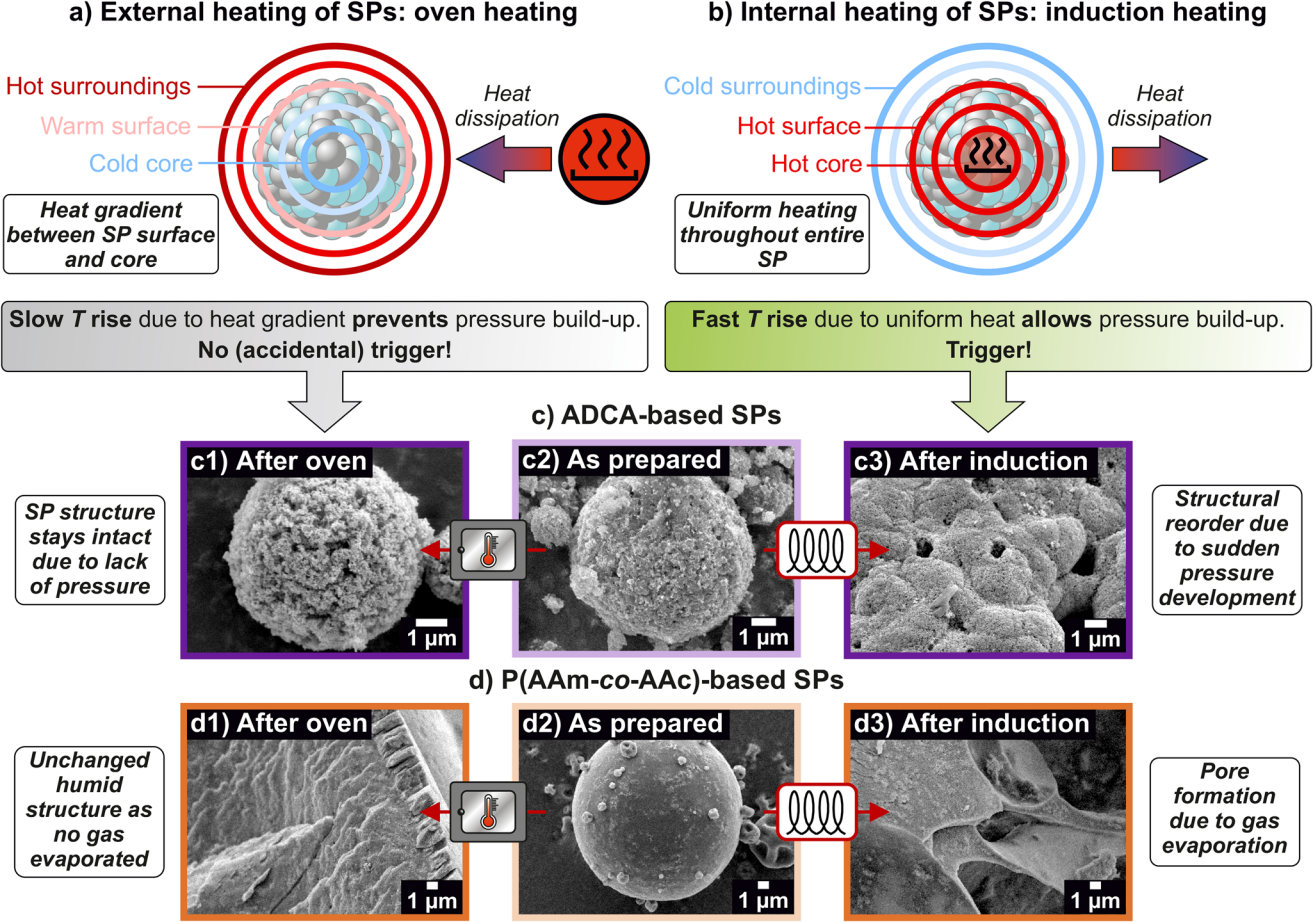
Comparison of global oven heating at 200°C for 1 h and induction heating for 10 s with regard to SP functionality. (a) External oven heating leads to heat dissipation from the surroundings to the material, resulting in heat gradients, a slow temperature rise, and therefore no successful trigger. (b) Internal induction heating leads to heat dissipation from the material to the surroundings, resulting in uniform heating, a fast temperature rise, and therefore a successful trigger. (c) In ADCA‐based SPs (c2), oven heating mediates an intact SP struc`ure due to the lack of pressure (c1), whereas induction heating causes a sudden pressure development resulting in structural reordering (c3). (d) In P(AAm‐*co*‐AAc)‐based SPs (d2), oven heating of the humidity‐treated state does not lead to structural changes, as no gas is produced and only non‐ionically bound water is evaporated (d1), whereas in induction heating, primary bound water is evaporated, leading to pore generation (d3).

When considering the energy consumption of both herein presented SP systems, only 0.7 (ADCA‐based) to 2.8 Wh (P(AAm‐*co*‐AAc)‐based) are required to induce effective expansion during induction heating, due to short needed impulse times of 1 to 5 s, respectively. In contrast, conventional oven processes are known to consume energy of up to 800 Wh [98], which is 300‐ to 1100‐fold of the total energy consumed during induction heating, further underlining the energetic benefit of the latter process.

Comparing both heating methods with regard to the presented functionality reveals that oven heating leads to a slow temperature increase that prevents the rapid pressure build‐up required to trigger either mechanism. Consequently, both SP model systems remain largely inactive under these conditions. For ADCA‐based SPs, oven treatment at 200°C for 1 h preserves the overall SP structure because the necessary pressure for restructuring does not develop. Although the temperature is sufficient to decompose ADCA, the produced gas diffuses slowly out of the SP instead of causing an instantaneous burst, resulting in loss of the ADCA component without structural reordering (Figures 5c1 vs. 5c2). Similarly, humidity‐treated P(AAm‐*co*‐AAc)‐based SPs show no structural change upon oven heating (Figure 5d1), as external heating primarily dries the particles rather than inducing sufficiently fast internal vaporization, and pressure build‐up is therefore absent. In contrast, induction heating allows a rapid internal temperature rise, enabling confined pressure development that triggers the respective functionalities. For ADCA‐based SPs, this results in the known structural reordering caused by sudden gas release (Figure 5c3), while in the P(AAm‐*co*‐AAc) system internal vapor pressure leads to extensive pore formation and expansion (Figure 5d2). This selective activation by rapid internal heating represents a key advantage of the concept, as accidental triggering by gradual external temperature increases is effectively prevented and functionality is exerted exclusively upon AMF exposure.

However, the external heat has different effects on the functionality depending on the system in focus. In the ADCA system, oven heat can deactivate the material if the chosen temperature is too high in the sense that an inductive trigger afterwards is not possible anymore, due to decomposition of ADCA within the SP structure. It has been shown that the SP can withstand temperatures of 100°C for 1 h and retain functionality, however, it becomes limited at 150°C and completely canceled out at 200°C (see Figure S19a–c). For the P(AAm‐*co*‐AAc) system, functionality is maintained at significantly higher oven temperatures (up to 200°C) for 1 h due to the working mechanism during which the polymer shell dries with global heating (see Figure S19d–f). However, it should be noted that the external temperature should not exceed the limit at which the polymer decomposition sets in.

For sufficient operation of the proposed mechanism, it is important to not only consider oven vs. induction heating, but also the induction heating rate of the employed magnetic NPs in themselves. If NPs with a slow heating rate are utilized, the produced gas in both cases has enough time to dissipate out of the SP structure, which prevents the needed pressure build‐up; additionally, lower temperatures are reached altogether due to the faster heat dissipation to the surroundings (see Figure S20a1). In contrast, a high heating rate ensures that enough gas is produced on a short time scale, and this fast gas generation leads to sufficient pressure development (see Figure S20a2). To showcase this, Co ferrite (CoFe_2_O_4_) NPs are used instead of FIONs, which show a significantly lower heating rate, but also considerably high Curie temperature around 350°C [24]. As a result, the trigger in the ADCA‐based system is reached, but to a lower effect due to gas dissipation (see Figure S20b). Contrarily, no trigger is reached in the P(AAm‐*co*‐AAc)‐based system, as the gas and heat dissipation in the model ratio of 8:2 is too large, hence preventing volume expansion altogether (see Figure S20c).

Accessing the functional response in both systems thus requires localized induction heating rather than global heat treatment. Importantly, not only the absolute temperature but also the heating rate of the magnetic NPs critically determine system performance. The desired functionality is obtained only within a narrow heating regime, while deviations in heating modality or rate lead to incomplete activation or loss of functionality altogether. This behavior indicates that the underlying processes are governed by kinetically controlled, localized energy dissipation, which cannot be replicated by homogeneous bulk heating. While this enables a high degree of control, it simultaneously represents a significant experimental challenge.

### 2.6 Proof of Principle for Application‐Oriented Scenarios

After elucidating the working mechanisms and thermal responses of both inductively triggerable SP systems, their ability to convert localized heating into mechanical force is evaluated in an application‐relevant scenario. Specifically, the SPs are integrated into a rigid matrix to assess whether the expansion‐induced stresses generated on the microscale are sufficient to overcome the mechanical resistance of the surrounding material. Therefore, the triggerable (if necessary pre‐treated) SP systems are incorporated between two layers of epoxy resin, which are colored differently for visual distinction and subsequently fully cured (Figure 6a1). Epoxy resin is selected as a representative matrix as conventional thermosetting epoxies form permanent three‐dimensional covalent networks upon curing, which conventionally cannot be reshaped or dissolved without irreversible bond scission [99, 100, 101]. In contrast to dynamic covalent networks such as vitrimers, which require global heating to enable bond exchange and network rearrangement [102, 103], these static networks exhibit intrinsically restricted chain mobility. As such, epoxy provides a stringent model system for probing whether microscale pressure generation from magnetically triggered expansion can produce localized mechanical stresses sufficient to disrupt a permanent thermoset matrix and thereby initiate controlled separation.

**FIGURE 6:**
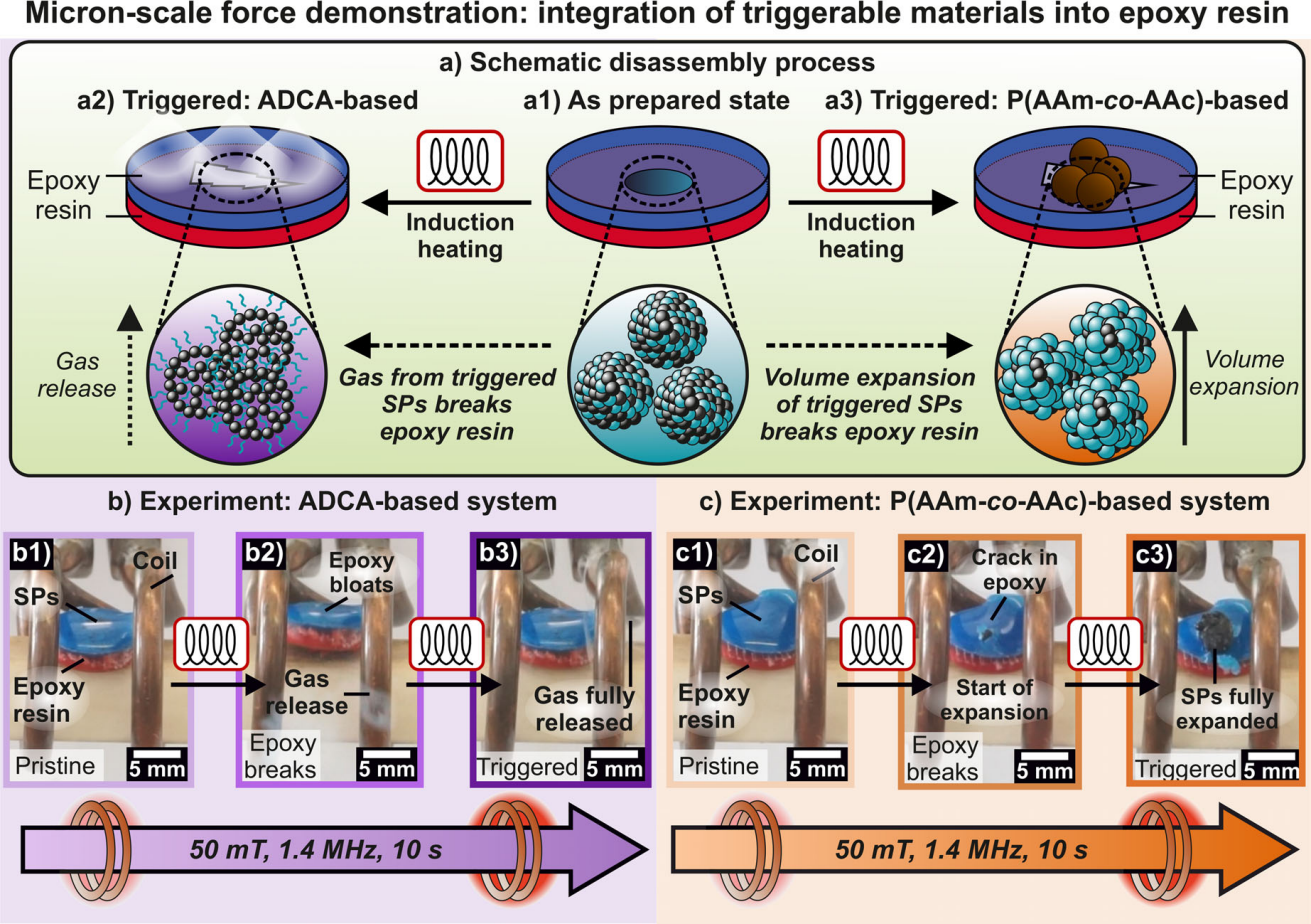
Proof of concept that the generated forces of triggerable SPs are sufficient to break even epoxy resins. (a) Schematic representation of the opening process with SPs embedded within epoxy resin: (a1) as prepared state, (a2) inductively triggered ADCA‐based model system, in which gas is released from the cracked epoxy resin, and (a3) inductively triggered P(AAm‐*co*‐AAc) model system that is broken by volume expansion within the epoxy. (b) Experimental triggering of the ADCA‐based system, in which the trigger leads to epoxy bloating (b2) and breaking (b3) for complete gas release within 10 s. (c) Experimental triggering of the P(AAm‐*co*‐AAc)‐based system, in which the trigger leads to volume expansion cracking (c2) and consequently breaking the epoxy (c3) within 10 s.

In the encapsulated ADCA‐based epoxy (Figure 6b1), induction heating leads to gas formation within the SPs embedded in the matrix. The developed gas is released from the SPs, making the epoxy bloat due to gas accumulation inside, before the pressure gets too high and the gas is released through an initial crack, making the epoxy break (Figure 6b2). In the triggered state, the gas is fully removed through the formed cracks in the epoxy resin, as pressure can no longer build up and gas is dissipated easily (Figures 6b3 and 6a2). Hence, the epoxy resin is successfully broken by the gas‐generating system upon the induction trigger within 10 s, relating to a total energy consumption of still only 7.4 Wh (corresponding video available online, Supplementary Video S9).

For encapsulated P(AAm‐*co*‐AAc)‐based materials (Figure 6c1), the start of the induction trigger leads to volume expansion of the SPs embedded in the matrix, resulting in a crack in the epoxy already at the start of the expansion process (Figure 6c2). This is due to large developed forces that surpass the cohesive forces of the epoxy. In the triggered state, the volume expansion further progresses, subsequently forming a large crack in the epoxy as the material can fully unfold and break the resin due to the developed pressure (Figure 6c3). Thus, also for this system, the epoxy is successfully broken through the induction trigger within 10 s (corresponding video is available online, Supplementary Video S10).

Repeated epoxy cracking experiments (*n* = 4) resulted in successful disruption in all cases, indicating reproducible force generation by both SP systems (Figure S21a). Optical microscopy showed that cracks consistently originated at the expandable particle layer and propagated through the bulk epoxy matrix (Figure S21b). No additional pores or gas defects were observed outside the induced crack. Exemplary Berkovich measurements of an epoxy inductively opened by ADCA:FION 1:1 SPs at the outside of the sample and near the crack revealed largely unchanged hardness and modulus values (0.15–0.18 and 3.2–3.6 GPa, respectively), indicating that extensive thermal softening of the surrounding epoxy matrix did not occur (Table S4). All indents showed thermal drift rates below 0.1 nm s^−^^1^ and were therefore considered valid for analysis. Together, these observations suggest that epoxy disruption is primarily driven by localized stress generation and internal pressure buildup at the expandable particle layer rather than by random defects, poor interfacial adhesion, or bulk thermal degradation of the epoxy matrix.

Summarizing, both systems enable rapid epoxy opening within 10 s of activation. Remarkably, this macroscopic response is achieved through initial gas generation leading to two fundamentally different mechanisms based on initial gas production: rapid gas release due to ADCA decomposition in one case and internal pressure build‐up within the SPs leading to bulk volume expansion in the other. Despite the distinct working principles, the response times are comparable, highlighting that fast pressure generation can be realized through multiple orthogonal actuation modes. The results therefore demonstrate a versatile and unusually rapid approach to generate large forces on the microscale, exemplarily demonstrated by epoxy opening.

## 3 Conclusion

We demonstrated the inductively triggered actuation of two distinct SP systems that exhibit their functionality toward micron‐scale force generation either by gas release or volumetric expansion, enabling flexibility in application scenarios ranging from gas‐tight matrices to rigid structures. For this, magnetic NPs—high‐heating‐rate FIONs or ZnCo ferrites with a controlled Curie temperature—were co‐spray‐dried with functional blowing agents, either ADCA or the superabsorbent polymer P(AAm‐*co*‐AAc). In ADCA‐based SPs, induction heating induces rapid thermal decomposition of ADCA into gaseous products, leading to rapid and burst‐like gas release on the macroscopic scale connected with structural reorganization on the SP level. In contrast, P(AAm‐*co*‐AAc)‐based SPs undergo controlled humidity and oven treatment after spray‐drying to enable water uptake within the superabsorbent network while forming a gas‐tight shell. Upon induction heating, vaporization of the encapsulated water generates internal pressure build‐up that expands the shell, producing large pores on the microscale and significant volume expansion on the macroscale.

In both systems, the maximum attainable temperature can be adjusted via the magnetic NP composition, while the trigger response can be tuned through the NP‐to‐blowing agent ratio, both variations easily adapted by the spray‐drying route. Notably, the desired functionality is exclusively achieved under induction heating, whereas conventional oven treatment fails to generate sufficient internal pressure. Both systems demonstrate remarkable mechanical performance, lifting up to 25 000 times their own mass within seconds. As a proof of concept, integration into epoxy resin matrices enabled rapid opening of cured resins within 10 s, demonstrating their potential for debonding and disassembly on demand applications.

The presented strategy establishes a general framework for magnetically responsive micrometer‐sized SP additives capable of converting localized electromagnetic energy into mechanical work on the micrometer scale. The experimental results demonstrate that distinct pressure‐generating mechanisms can be integrated with magnetic NPs to achieve rapid induction‐triggered actuation and substantial force generation that are not accessible by global heating. Furthermore, proof‐of‐concept experiments in cured epoxy matrices show that this actuation can be harnessed to induce structural opening within seconds upon magnetic activation. From a materials perspective, the concept is expected to be scalable, as increasing the amount of incorporated SPs proportionally increases the total heat generation capacity while maintaining the predefined ratio of magnetic NPs and blowing agent within each SP. Future implementation in engineering‐scale systems will, however, require consideration of factors such as heat dissipation, component geometry, and magnetic field distribution to ensure homogeneous activation throughout larger structures. While the present study is limited to proof‐of‐concept demonstrations, the underlying principle of remotely converting electromagnetic energy into localized pressure generation and mechanical work may provide a basis for future investigations into debonding systems, adaptive composites, reconfigurable structures, and other dynamic material concepts. More broadly, the ability to encode programmable mechanical functionality into particulate building blocks offers a versatile platform for designing materials that can be activated on demand to undergo controlled structural transformations.

## 4 Experimental Section

### 4.1 Materials and Reagents

Iron(II) sulfate heptahydrate (FeSO_4_·7 H_2_O, 99.5%), potassium nitrate (KNO_3_, 99+%), and citric acid monosodium salt (NaC_6_H_7_O_7_, 99%) were obtained from Acros Organics. Potassium hydroxide (KOH, 90%, flakes), iron(III) nitrate nonahydrate (Fe(NO_3_)_3_·9 H_2_O, 96%), cobalt(II) nitrate hexahydrate (Co(NO_3_)_2_·6 H_2_O, 98%), zinc(II) nitrate hexahydrate (Zn(NO_3_)_2_·6 H_2_O, 98%), sodium hydroxide (NaOH, 98%), and sodium bicarbonate (NaHCO_3_, 99+%) were received from Carl Roth. Tetraethyl orthosilicate (SiC_8_H_20_O_4_, TEOS, 99%) was obtained from abcr. Dimethyl sulfoxide (DMSO, 99.9%) was bought from Fisher Scientific. Poly(acrylamide‐*co*‐acrylic acid) (partial sodium salt, P(AAm‐*co*‐AAc), M_W_ = 520 000, M_n_ = 150 000, acrylamide ∼ 80 wt%) and azodicarbonamide (NH_2_CON = NCONH_2_, ADCA, 97%) were bought from Sigma Aldrich. Epoxy resin and dyes were obtained from Let's Resin. All chemicals and reagents were used without further purification. Water was deionized prior to usage.

### 4.2 Synthesis of FION NPs

FIONs were synthesized as per an oxidative precipitation method formerly introduced by our group [65]. FeSO_4_·7 H_2_O (10.01 g, 36.01 mmol) was dissolved in 1620 g of deionized H_2_O and heated to reflux under degassing with nitrogen. In a separate beaker, the precipitating agent consisting of KNO_3_ (25.48 g, 252.02 mmol) and KOH (6.17 g, 109.96 mmol) was dissolved in 180 g of deionized water, further degassed with nitrogen and heated to 60°C, before adding it to the reaction solution. The reaction mixture was left to boil for 5 h under reflux cooling and degassing with nitrogen, creating a dark brown suspension. Afterwards, the mixture was cooled down to room temperature, before the sedimented NPs were purified by centrifugation (3 × 8000 rpm for 2 min) and redispersed in 100 mL of deionized water to create an aqueous dispersion of unfunctionalized FIONs. For surface functionalization with citric acid, 1 g of FIONs was adjusted to a total volume of 300 mL in deionized water. Then, citric acid monosodium salt (90.00 mg, 0.42 mmol) was added, before the mixture was sonicated in an ultrasonic bath (Sonorex Super RK 514) for 15 min. For washing the citric acid‐functionalized FIONs, the reaction mixture was purified by centrifugation (3 × 12000 rpm for 5 min) prior to redispersion in 50 mL of deionized water. For redispersion of FIONs in DMSO, unfunctionalized FIONs in the desired amount were sedimented by centrifugation (1 × 8000 rpm, 2 min) and washed twice with DMSO in the centrifuge (2 × 8000 rpm, 2 min), before they were redispersed in DMSO.

### 4.3 Synthesis of ZnCoFe_2_O_4_ NPs

ZnCo ferrites were synthesized based on a modified spray‐drying method to obtain iron oxides introduced by Jo et al. [104] that was previously introduced by our group [24]. The molar ratio of precursors (Fe, Co, Zn) to Si was maintained at 0.4. Fe(NO_3_)_3_·9 H_2_O (3.93 g, 9.73 mmol), Co(NO_3_)_2_·6 H_2_O (0.85 g, 2.92 mmol), and Zn(NO_3_)_2_·6 H_2_O (0.58 g, 1.95 mmol) were dissolved in a mixture of H_2_O (21.62 g, 1.20 mol) and EtOH (27.64 g, 0.60 mol). TEOS (7.60 g, 36.48 mmol) was added dropwise to the stirred solution under a color change from red to orange–yellow. The precursor mixture was spray‐dried to yield a light yellow powder (yield ∼ 4 g) using a Büchi Labortechnik AG spray‐dryer (B‐290 mini) connected to a dehumidifier B‐296. Spray‐drying was conducted with the following parameters: inlet temperature 220°C, pump rate ∼ 0.18 L h^−1^, aspirator power 100%, resulting in a volume flow of ∼ 35 m^3^ h^−1^, spraying gas flow 742 L h^−1^, outlet temperature ∼ 100°C). After spray‐drying, the powder was heated at 1100°C for 4 h in a high‐temperature furnace (Nabertherm LT 5/11 with B400/B410 Controller) at a heating ramp of 300 K h^−1^, inducing a color change to dark brown. The powder was subsequently treated with an aqueous solution of NaOH (5 M, 100 mL) at 70°C for 24 h to remove SiO_2_ residues, before the resulting dispersions were centrifuged (Hermle Z 326 K, 1 × 12000 rpm for 1 min, 2 × 12000 rpm for 5 min) and redispersed in 40 mL of deionized water. For redispersion of ZnCoFe_2_O_4_ in DMSO, the NPs were sedimented by centrifugation (1 × 8000 rpm, 2 min) and washed twice with DMSO in the centrifuge (2 × 8000 rpm, 2 min), before they were redispersed in DMSO (40 mL). CoFe_2_O_4_ NPs were synthesized in the same manner with Fe(NO_3_)_3_·9 H_2_O (5.31 g, 13.1 mmol) and Co(NO_3_)_2_·6 H_2_O (0.42 g, 1.46 mmol) as salt precursors.

### 4.4 Synthesis of P(AAm‐*co*‐AAc)‐Based SPs

P(AAm‐*co*‐AAc)‐based SPs were formed by spray‐drying aqueous dispersions of the corresponding iron oxide NP species with an aqueous solution of P(AAm‐*co*‐AAc) (5 wt%) using a lab‐scale spray‐dryer (Büchi Labortechnik AG, B‐290 mini, connected to a dehumidifier B‐296) under identical parameters in each experiment (inlet temperature: 85°C, pump rate: ∼ 0.18 L h^−1^, aspirator power: 85%, resulting in a volume flow of ∼ 33 m^3^ h^−1^, spraying gas flow: 473 L h^−1^, outlet temperature: ∼ 40°C). The components were mixed in the respective desired ratio under non‐magnetic stirring with an overhead stirrer, optionally together with NaHCO_3_, before they were spray‐dried employing a total particle concentration of 3.5 wt%. The obtained brown SP powders were dried at 40°C and 30 mbar in a vacuum oven (VO29, Memmert) for at least 8 h. To treat the powders with humidity afterwards, the SPs were placed in a climate chamber (Binder MKF‐56‐E5) at 30°C and 98% relative humidity for 4 h except explicitly stated otherwise. For an optional drying step post humidity exposure, the composites were dried in a vacuum oven (VO29, Memmert) at 100°C for 1 h except explicitly stated otherwise.

### 4.5 Synthesis of ADCA‐Based SPs

ADCA‐based SPs were synthesized by spray‐drying dispersions of the corresponding iron oxide species in DMSO with solutions of ADCA in DMSO (2 wt%) using a lab‐scale spray‐dryer (Büchi Labortechnik AG, B‐290 mini, connected to a dehumidifier B‐296) under identical parameters in each experiment (inlet temperature: 140°C, pump rate: ∼ 0.18 L h^−1^, aspirator power: 85%, resulting in a volume flow of ∼ 33 m^3^ h^−1^, spraying gas flow: 473 L h^−1^, outlet temperature: ∼ 80°C). For reference experiments, ADCA was dispersed in water (2 wt%) prior to spray‐drying together with aqueous dispersions of iron oxide NPs. The components were mixed in the respective desired ratio under non‐magnetic stirring with an overhead stirrer, before they were spray‐dried employing a total particle concentration of 2 wt%. The obtained brown SP powders were dried at 40°C and 30 mbar in a vacuum oven (VO29, Memmert) for at least 8 h.

### 4.6 Integration of SPs Into Epoxy Resin

Epoxy resin and hardener (ratio 1:1) were mixed together with red dye and statically stirred for 2 min, before applying to an aluminum pan of the desired form and leaving the mixture to harden overnight. SPs (20 mg) were placed in the middle of the dried resin, before viscous epoxy with blue dye was yet again placed on top of the composites. The mixture was again dried overnight to harden the epoxy resin, before the aluminum pan was manually removed, revealing epoxy‐encapsulated SPs that were further placed in the center of the coil to be inductively heated.

### 4.7 Material Characterization

Scanning electron microscopy (SEM) characterization was conducted using a JSM‐F100 (Jeol) with an acceleration voltage of 5 kV and a working distance of 6 mm. Samples were prepared on carbon pads (Plano). All samples were sputtered with Pt (108SE, Cressington, 40 mA, 20 s).

Induction heating experiments were conducted employing the high‐frequency device Sinus 52 (Himmelwerk Hoch‐ und Mittelfrequenzanlagen GmbH) with a water‐cooled coil consisting of two turns of copper tubing (outer diameter: 30 mm, inner diameter: 22 mm). Samples in a crucible were positioned centrally between the two turns of the induction coil (pitch: 10 mm) in an alumina crucible (75 µL, outer diameter: 6 mm) on an alumina combustion boat (height: 9 mm), concluding a coil‐to‐sample distance of 2 mm on both sides. Samples in a syringe (diameter: 5 mm) were placed centrally within the coil, with a consequent coil‐to‐sample distance of 8.5 mm on both sides. Experiments were conducted at a frequency of 1419 kHz at an environmental humidity of 29%. The AMF amplitude at the sample position was obtained from a manufacturer‐provided calibration of the induction system for the specific coil geometry used in this study, with the maximum amplitude of 50 mT taken from the simulation at the sample position. For the induction heating of dried NP and spray‐dried SP powder samples, 30 mg (20 mg for ADCA‐based SPs) of the respective sample were placed in the center of the coil. The temperature at the surface of magnetic samples was measured with a CTlaser LTF pyrometer (Optris). The pyrometer emissivity was set to 0.97 with the measurement accuracy being ±1% of the reading or ±1°C, whichever is greater, as given by the manufacturer. For measurements in a syringe, 20 mg of the corresponding sample were transferred to a 1 mL syringe (Injekt, B. Braun) that was manually cut and sealed at the bottom, before induction heating was triggered in the center of the coil. The frictional force between the syringe and plunger was determined from a dead‐weight method to be 0.54 N, corresponding to a pressure of 0.275 bar. The electrical energy consumption of the induction heating system was monitored using a Shelly Pro 3EM three‐phase energy meter (Shelly Group, Sofia, Bulgaria). The device was connected to all three supply phases of the induction heater and used to record voltage, current, power, and cumulative energy consumption during each experiment. The total electrical energy input was obtained by summing the energy consumption recorded for the three phases. The cumulative energy consumption (Wh) measured by the Shelly Pro 3EM was used to calculate the total electrical energy input for the determination of the electrical‐to‐mechanical energy conversion efficiency.

Magnetization curves of magnetic NP and SP samples were obtained using a superconducting quantum interference device (SQUID) MPMS3 (Quantum Design Inc.). The corresponding curves were obtained by cycling the applied magnetic field from −3 to 3 T employing a step rate of 5 mT s^−1^. The temperature for all measurements was set to 300 K.

Transmission electron microscopy (TEM) images were recorded with a LEO EM 900 microscope (Carl Zeiss AG, Oberkochen, Germany) using an acceleration voltage of 80 kV. Samples were prepared on copper TEM grids with carbon support films. NP size distributions were averaged over 100 NPs from TEM images by manual evaluation using ImageJ.

Thermogravimetric analysis (TGA) measurements were conducted on 5–10 mg of SP powder on a TG 209 F1 Libra (Netzsch) by heating the samples in synthetic air with a heating rate of 10 K min^−1^ in the temperature range from 30 to 1000°C.

Magnetic particle spectroscopy (MPS) was performed with an MPS unit (PureDevices GmbH) in a sinusoidal alternating magnetic field of 20 kHz in a range of ± 30 mT at 37°C. For each sample, three measurements were taken at each corresponding state, for which the materials were placed in a cylindrical glass vial that fit into the MPS unit. The displayed magnetization curves represent the mean average of 20 measurements.

Attenuated total reflection infrared (ATR‐IR) spectroscopy was carried out using an IRTracer‐100 with a QATR 10 Single‐Reflection ATR Accessory with a diamond crystal (Shimadzu). Background‐corrected spectra were recorded between 500 and 4000 cm^−1^ with a resolution set to 1 cm^−1^.

Optical microscopy analysis was performed using the trinocular zoom stereo microscope VisiScope SZT360‐6 (VWR) connected to the microscopy camera Flexacam c5 (Leica).

The mechanical properties of epoxy resin samples were evaluated using an Agilent Nanoindenter XP equipped with a Berkovich indenter tip. Nanoindentation was performed to a maximum indentation depth of 1000 nm at a constant indentation strain rate of 0.025 s^−^^1^. Hardness and elastic modulus were calculated according to the method proposed by Oliver and Pharr [105], using a Poisson's ratio of 0.35. The reported values correspond to averages obtained over an indentation depth range of 500–950 nm. The reported results represent the average of four measurements taken at the epoxy edge and four measurements acquired in the vicinity of the crack, respectively.

Videos were recorded with a digital camera (Alpha6000, Sony) with a frame rate of 25 fps, while snapshots of the videos were taken at characteristic times during the induction heating procedure. The onset time of the trigger function was determined by the time stamp of the corresponding videos.

## Author Contributions

L. Luthardt (Conceptualization: Lead; Investigation: Lead; Methodology: Lead; Data Curation: Lead; Funding acquisition: Equal; Supervision: Lead; Validation: Lead; Visualization: Lead; Writing – original draft: Lead; Writing – review & editing: Lead), S. Müssig (Conceptualization: Equal; Methodology: Equal; Supervision: Equal, Writing – review & editing: Equal; Visualization: Equal), R. Luxenhofer (Conceptualization: Equal; Writing – review & editing: Equal), K. Mandel (Conceptualization: Lead; Funding acquisition: Lead; Project administration: Lead; Resources: Lead; Supervision: Equal, Writing – review & editing: Equal).

## Funding

Federal Ministry of Research, Technology and Space (Bundesministerium für Forschung, Technologie und Raumfahrt, BMFTR, AdRecBat No. 03XP0518C), Deutsche Forschungsgemeinschaft (DFG, German Research Foundation, Project‐ID – 416229255, CRC1411), European Union (ERC, SmartRust, 101123921), German Federal Environmental Foundation (Deutsche Bundesstiftung Umwelt, DBU).

## Conflicts of Interest

The authors declare no conflicts of interest.

## Supporting information




**Supporting File 1**: adma74932‐sup‐0001‐SuppMat.docx.

**Supporting File 2**:adma74932‐sup‐0002‐VideoS1.mp4.

**Supporting File 3**: adma74932‐sup‐0003‐VideoS2.mp4.

**Supporting File 4**: adma74932‐sup‐0004‐VideoS3.mp4.

**Supporting File 5**: adma74932‐sup‐0005‐VideoS4.mp4.

**Supporting File 6**:adma74932‐sup‐0006‐VideoS5.mp4.

**Supporting File 7**: adma74932‐sup‐0007‐VideoS6.mp4.

**Supporting File 8**: adma74932‐sup‐0008‐VideoS7.mp4.

**Supporting File 9**: adma74932‐sup‐0009‐VideoS8.mp4.

**Supporting File 10**: adma74932‐sup‐0010‐VideoS9.mp4.

**Supporting File 11**: adma74932‐sup‐0011‐VideoS10.mp4.

## Data Availability

The data that supports the findings of this study is openly available on Zenodo https://doi.org/10.5281/zenodo.21977806.

## References

[1] S. Nocentini , C. Parmeggiani , D. Martella , and D. S. Wiersma , “Optically Driven Soft Micro Robotics,” Advanced Optical Materials 6, no. 14 (2018): 1800207, 10.1002/adom.201800207.

[2] G. T. Iványi , B. Nemes , I. Gróf , et al., “Optically Actuated Soft Microrobot Family for Single‐Cell Manipulation,” Advanced Materials 36, no. 32 (2024): 2401115, 10.1002/adma.202401115. 38814436

[3] Q. Ji , J. Moughames , X. Chen , et al., “4D Thermomechanical Metamaterials for Soft Microrobotics,” Communications Materials 2, no. 1 (2021): 93, 10.1038/s43246-021-00189-0.

[4] Z. Wang , J. Wang , J. Ayarza , et al., “Bio‐inspired Mechanically Adaptive Materials Through Vibration‐induced Crosslinking,” Nature Materials 20, no. 6 (2021): 869–874, 10.1038/s41563-021-00932-5. 33619367

[5] R. Xu , C. Linghu , W. Mao , et al., “Shape‐Adaptive Mechanical Metastructure Enables Robust Adhesion and Dynamic Capturing of 3D Objects,” Advanced Functional Materials 36, no. 6 (2026): 14499, 10.1002/adfm.202514499.

[6] P. Feng , R. Zhao , W. Tang , et al., “Structural and Functional Adaptive Artificial Bone: Materials, Fabrications, and Properties,” Advanced Functional Materials 33, no. 23 (2023): 2214726.

[7] H. L. Wang , T. Chen , B. Zhang , et al., “A Dual‐Responsive Artificial Skin for Tactile and Touchless Interfaces,” Small 19, no. 21 (2023): 2206830, 10.1002/smll.202206830. 36700923

[8] J. Yi , G. Zou , J. Huang , et al., “Water‐responsive Supercontractile Polymer Films for Bioelectronic Interfaces,” Nature 624, no. 7991 (2023): 295–302, 10.1038/s41586-023-06732-y. 38092907

[9] S. Stock , L. Mirau , M. Rutsch , et al., “Ultrasound‐Induced Adsorption of Acousto‐Responsive Microgels at Water‐Oil Interface,” Advanced Science 11, no. 5 (2024): 2305395. 10.1002/advs.202305395 PMC10837341 38093508

[10] Y. Xu , Q. Bian , R. Wang , and J. Gao , “Micro/Nanorobots for Precise Drug Delivery via Targeted Transport and Triggered Release: a Review,” International Journal of Pharmaceutics 616 (2022): 121551, 10.1016/j.ijpharm.2022.121551. 35131352

[11] A. Sneider , D. VanDyke , S. Paliwal , and P. Rai , “Remotely Triggered Nano‐Theranostics for Cancer Applications,” Nanotheranostics 1, no. 1 (2017): 1–22, 10.7150/ntno.17109. PMC5298883 28191450

[12] M. A. M. Munshi , E. H. Chowdhury , L. E. Paniagua‐Guerra , J. Dionne , A. Giri , and B. Ramos‐Alvarado , “A Review of Heat Transport in Solvated Gold Nanoparticles: Molecular Dynamics Modeling and Experimental Perspectives,” Nanoscale 17, no. 36 (2025): 20803–20830, 10.1039/D5NR02521D. 40889186

[13] Y. Hou , H. Wang , R. Fu , et al., “A Review on Microrobots Driven by Optical and Magnetic Fields,” Lab on a Chip 23, no. 5 (2023): 848–868, 10.1039/D2LC00573E. 36629004

[14] J. J. Huaroto and S. Misra , “Size and Illumination Matters: Local Magnetic Actuation and Fluorescence Imaging for Microrobotics,” Journal of the Indian Institute of Science 104, no. 3 (2024): 745–763, 10.1007/s41745-024-00453-5. PMC12069155 40370769

[15] M. Birla , J. Zou , Z. Afkhami , et al., “Multi‐photon 3D Imaging With an Electrothermal Actuator With Low Thermal and Inertial Mass,” Sensors and Actuators A: Physical 329 (2021): 112791, 10.1016/j.sna.2021.112791.

[16] S. Liu , H. Liang , and Y. Yin , “Site‐selective Reprogrammable Actuators for Soft Robotic Systems Using Plasmonic Photothermal Conversion,” Device 2, no. 4 (2024): 100330, 10.1016/j.device.2024.100330.

[17] Y. Chen and D. Lei , “Analysis and Numerical Simulation Research of the Heating Process in the Oven,” Journal of Thermal Science 25, no. 5 (2016): 454–459, 10.1007/s11630-016-0884-5.

[18] S. Mahdi , H.‐J. Kim , B. A. Gama , S. Yarlagadda , and J. W. Gillespie , “A Comparison of Oven‐cured and Induction‐cured Adhesively Bonded Composite Joints,” Journal of Composite Materials 37, no. 6 (2003): 519–542, 10.1177/0021998303037006776.

[19] E. C. Abenojar , S. Wickramasinghe , J. Bas‐Concepcion , and A. C. S. Samia , “Structural Effects on the Magnetic Hyperthermia Properties of Iron Oxide Nanoparticles,” Progress in Natural Science: Materials International 26, no. 5 (2016): 440–448, 10.1016/j.pnsc.2016.09.004.

[20] W. Jin , C. Xing , Y. Lu , S. Baoshou , and D. Li , “A Novel Method Improving the Temperature Uniformity of Hot‐plate Under Induction Heating,” Proceedings of the Institution of Mechanical Engineers, Part C: Journal of Mechanical Engineering Science 235, no. 1 (2021): 190–201.

[21] O. Lucia , P. Maussion , E. J. Dede , and J. M. Burdio , “Induction Heating Technology and Its Applications: Past Developments, Current Technology, and Future Challenges,” IEEE Transactions on Industrial Electronics 61, no. 5 (2014): 2509–2520, 10.1109/TIE.2013.2281162.

[22] P. Guardia , A. Labarta , and X. Batlle , “Tuning the Size, the Shape, and the Magnetic Properties of Iron Oxide Nanoparticles,” The Journal of Physical Chemistry C 115, no. 2 (2011): 390–396, 10.1021/jp1084982.

[23] A. A. de Almeida , F. Fabris , G. S. Da Silva , K. R. Pirota , M. Knobel , and D. Muraca , “Control of Anisotropy and Magnetic Hyperthermia Effect by Addition of Cobalt on Magnetite Nanoparticles,” ACS Applied Materials & Interfaces 17, no. 9 (2025): 13083–13093. 10.1021/acsami.4c03343 PMC11891862 39007483

[24] L. Luthardt , T. Raczka , K. Hurle , S. Müssig , and K. Mandel , “Customizable Induction Heating Profiles: From Tailored Colloidally Stable Nanoparticles Toward Multi‐Stage Heatable Supraparticles,” Advanced Functional Materials 35, no. 2 (2025): 2412296, 10.1002/adfm.202412296.

[25] Z. Nemati , J. Alonso , I. Rodrigo , et al., “Improving the Heating Efficiency of Iron Oxide Nanoparticles by Tuning Their Shape and Size,” The Journal of Physical Chemistry C 122, no. 4 (2018): 2367–2381, 10.1021/acs.jpcc.7b10528.

[26] U. Shankar , A. Kumar , S. K. Chaurasia , P. Kumar , F. A. Latif , and M. Z. A. Yahya , “Structural, Optical, and Magnetic Properties of PMMA‐Magnetite (Fe3O4) Composites: Role of Magneto‐Conducting Filler Particles,” Journal of Electronic Materials 52, no. 7 (2023): 4375–4387, 10.1007/s11664-023-10400-2.

[27] L. Paleari , G. Varvaro , F. Nanni , et al., “On the Use of Magnetite Nanoparticles as Filler for 3D Printing of Polyether‐Ether‐Ketone‐Based Soft Magnets,” Polymer Composites 46, no. 11 (2025): 10298–10315, 10.1002/pc.29622.

[28] S. I. Ahmed , Z. K. Heiba , M. M. Ibrahim , et al., “Synthesis and Characterization of Eco‐Friendly CMC/Maghemite Nanocomposite Films,” Journal of Electronic Materials 50, no. 12 (2021): 7098–7109, 10.1007/s11664-021-09237-4.

[29] G. W. Kim , S. Kim , and H. J. Choi , “Enhanced Performance of Nano‐sized Maghemite Added Carbonyl Iron‐based Magnetorheological Soft Elastomer,” Journal of Magnetism and Magnetic Materials 560 (2022): 169659, 10.1016/j.jmmm.2022.169659.

[30] Y. Cao , M. Zhu , M. Z. Rong , and M. Q. Zhang , “Injection Moldable, Self‐healable, and Recyclable Rubber‐bonded NdFeB Magnets With the Magnetic Particulates Content up to 90 Wt%,” Advanced Composites and Hybrid Materials 6, no. 1 (2023): 38, 10.1007/s42114-023-00623-6.

[31] Z. Wu , Q. Wang , J. Huang , et al., “The Soft NdFeB/Ecoflex Composites for Soft Robot With a Considerable Magnetostimulated Shrinkability,” Composites Science and Technology 217 (2022): 109129, 10.1016/j.compscitech.2021.109129.

[32] A. Avasthi , C. Caro , M. L. Garcia‐Martin , and M. P. Leal , “Deciphering the Parameters to Produce Highly Reproducible and Scalable Iron Oxide Nanoparticles,” Reaction Chemistry & Engineering 8, no. 7 (2023): 1638–1653, 10.1039/D2RE00516F.

[33] D. Ling and T. Hyeon , “Chemical Design of Biocompatible Iron Oxide Nanoparticles for Medical Applications,” Small 9, no. 9‐10 (2013): 1450–1466, 10.1002/smll.201202111. 23233377

[34] M. A. Yousefi , D. Rahmatabadi , M. Baniassadi , M. Bodaghi , and M. Baghani , “4D Printing of Multifunctional and Biodegradable PLA‐PBAT‐Fe 3 O 4 Nanocomposites With Supreme Mechanical and Shape Memory Properties,” Macromolecular Rapid Communications 46, no. 2 (2025): 2400661, 10.1002/marc.202400661. PMC11756866 39401289

[35] K. Mirasadi , M. A. Yousefi , L. Jin , et al., “4D Printing of Magnetically Responsive Shape Memory Polymers: Toward Sustainable Solutions in Soft Robotics, Wearables, and Biomedical Devices,” Advanced Science 13, no. 15 (2026): 13091. 10.1002/advs.202513091 PMC13042656 40847440

[36] M. A. Yousefi , D. Rahmatabadi , M. Ahmadi , et al., “4D Printing for Minimally Invasive Biomedical Applications: Programmable Smart Materials for Deployable Devices, Drug Delivery, and Tissue Regeneration,” Materials & Design 263 (2026): 115555, 10.1016/j.matdes.2026.115555.

[37] Y. Li , Y. Tie , and C. Li , “Magneto‐thermally Triggered Self‐healing in Fe_3_O_4_/EMAA Composites: Mechanical Reinforcement and Induction Heating Performance,” Composites Communications 63 (2026): 102767, 10.1016/j.coco.2026.102767.

[38] B. Oberhausen and G. Kickelbick , “Induction Heating Induced Self‐healing of Nanocomposites Based on Surface‐functionalized Cationic Iron Oxide Particles and Polyelectrolytes,” Nanoscale Advances 3, no. 19 (2021): 5589–5604, 10.1039/D1NA00417D. PMC9417805 36133272

[39] X. Cheng , Y. Zhou , A. D. M. Charles , et al., “Enabling Contactless Rapid On‐demand Debonding and Rebonding Using Hysteresis Heating of Ferrimagnetic Nanoparticles,” Materials & Design 210 (2021): 110076, 10.1016/j.matdes.2021.110076.

[40] M. Kanidi , N. Loura , A. Frengkou , T. K. Milickovic , A.‐F. Trompeta , and C. Charitidis , “Inductive Thermal Effect on Thermoplastic Nanocomposites With Magnetic Nanoparticles for Induced‐Healing, Bonding and Debonding on‐Demand Applications,” Journal of Composites Science 7, no. 2 (2023): 74, 10.3390/jcs7020074.

[41] G. P. Mendes , L. D. Kluskens , M. Mota , S. Lanceros‐Méndez , and T. A. Hatton , “Spherical and Needle Shaped Magnetic Nanoparticles for Friction and Magnetic Stimulated Transformation of Microorganisms,” Nano‐Structures & Nano‐Objects 26 (2021): 100732, 10.1016/j.nanoso.2021.100732.

[42] J. Tang , Q. Yin , M. Shi , et al., “Programmable Shape Transformation of 3D Printed Magnetic Hydrogel Composite for Hyperthermia Cancer Therapy,” Extreme Mechanics Letters 46 (2021): 101305, 10.1016/j.eml.2021.101305.

[43] T. Yan , D. Rao , Y. Chen , Y. Wang , Q. Zhang , and S. Wu , “Magnetic Nanocomposite Hydrogel With Tunable Stiffness for Probing Cellular Responses to Matrix Stiffening,” Acta Biomaterialia 138 (2022): 112–123, 10.1016/j.actbio.2021.11.001. 34749001

[44] F. Li , Q. Zhang , S. Jiao , et al., “Preparation, Characterization and Foaming Performance of Thermally Expandable Microspheres,” Materials Research Express 7, no. 11 (2020): 115302, 10.1088/2053-1591/abc4b1.

[45] J. C. Gomez , N. S. Vishnosky , J. T. Grafstein , S. T. Kim , and R. C. Steinhardt , “Thermally Expandable Microspheres With High Expansion Ratios: Design of Core and Shell for Largest Size Change,” Journal of Applied Polymer Science 139, no. 28 (2022): 52517, 10.1002/app.52517.

[46] J.‐B. Desbrest , P. Garrabos , M. D. Wakeman , et al., “Design of Debondable Composite Sandwich Structures Containing a Primer Based on Thermally Expandable Microspheres: Process Window & Thermomechanical Performance Evaluation,” Composites Part A: Applied Science and Manufacturing 208 (2026): 109922, 10.1016/j.compositesa.2026.109922.

[47] F. Wanghofer , A. Wolfberger , G. Oreski , L. Neumaier , and S. Schlögl , “Investigation of Expandable Fillers for Reversible Adhesive Bonding in Photovoltaic Modules,” International Journal of Adhesion and Adhesives 126 (2023): 103454, 10.1016/j.ijadhadh.2023.103454.

[48] H. T. Kim , A. K. Jaladi , Y. J. Lee , and D. K. An , “Thermal Expansion Behavior of Thermally Expandable Microspheres Prepared by Suspension Polymerization Using P(AN‐MMA‐MAA) Core/Shell,” Bulletin of the Korean Chemical Society 41, no. 2 (2020): 190–195, 10.1002/bkcs.11952.

[49] B. Salahuddin , H. Warren , and G. M. Spinks , “Thermally Actuated Hydrogel Bead Based Braided Artificial Muscle,” Smart Materials and Structures 29, no. 5 (2020): 055042, 10.1088/1361-665X/ab79ba.

[50] H. Choudhary and S. R. Raghavan , “Superfast‐Expanding Porous Hydrogels: Pushing New Frontiers in Converting Chemical Potential Into Useful Mechanical Work,” ACS Applied Materials & Interfaces 14, no. 11 (2022): 13733–13742, 10.1021/acsami.2c00645. 35261243

[51] J. Ye , W. Dong , Q. Liao , et al., “Effect of Foaming Agent Types and Content on Polylactic Acid Foam Materials Prepared by Fused Deposition Modeling,” Polymers for Advanced Technologies 36, no. 12 (2025): 70448, 10.1002/pat.70448.

[52] W. Gong , X. Pei , X. Yin , D. Ban , H. Fu , and L. He , “Synthesis of High‐temperature Thermally Expandable Microcapsules and Their Effects on Foaming Quality and Surface Quality of Foamed ABS Materials,” e‐Polymers 20, no. 1 (2020): 519–527.

[53] Y. Wang , Z. Hu , H. Li , W. Xu , and J. Liu , “Expansive Deformation and Mechanical Properties of Cementitious Materials With Azodicarbonamide Expansive Agent: Influencing Factors and Mechanisms,” Journal of Sustainable Cement‐Based Materials 12, no. 9 (2023): 1141–1155, 10.1080/21650373.2023.2175272.

[54] E. Loiseau , A. Q. de Boiry , F. Niedermair , G. Albrecht , P. A. Rühs , and A. R. Studart , “Explosive Raspberries: Controlled Magnetically Triggered Bursting of Microcapsules,” Advanced Functional Materials 26, no. 22 (2016): 4007–4015, 10.1002/adfm.201504656.

[55] J. Ogbebor , S. A. Flores Roman , G. Jomon , and G. Y. Gor , “Compressibility of Confined Fluids From Volume Fluctuations,” Langmuir 41, no. 51 (2025): 34189–34196, 10.1021/acs.langmuir.5c03971. PMC12756912 41397103

[56] F. Keyvani , A. Safaei , Y. Kazemzadeh , M. Riazi , and J. Qajar , “Impact of Nanopore Confinement on Phase Behavior and Enriched Gas Minimum Miscibility Pressure in Asphaltenic Tight Oil Reservoirs,” Scientific Reports 14, no. 1 (2024): 13405, 10.1038/s41598-024-64194-2. PMC11167058 38862707

[57] W. Lauterborn , T. Kurz , R. Mettin , and C. D. Ohl , “Experimental and Theoretical Bubble Dynamics,” Advances in Chemical Physics 110 (2007): 295–380.

[58] S. Wintzheimer , T. Granath , M. Oppmann , et al., “Supraparticles: Functionality From Uniform Structural Motifs,” ACS Nano 12, no. 6 (2018): 5093–5120, 10.1021/acsnano.8b00873. 29763295

[59] S. Wintzheimer , L. Luthardt , K. L. A. Cao , et al., “Multifunctional, Hybrid Materials Design via Spray‐Drying: Much More Than Just Drying,” Advanced Materials 35, no. 47 (2023): 2306648, 10.1002/adma.202306648. 37840431

[60] H. Caglar , Y. A. Aksoy , S. Idapalapati , B. Caglar , M. Sharma , and C. K. Sin , “Debonding‐on‐demand Fe 3 O 4 ‐epoxy Adhesively Bonded Dissimilar Joints via Electromagnetic Induction Heating_3_O_4_‐epoxy Adhesively Bonded Dissimilar Joints via Electromagnetic Induction Heating,” The Journal of Adhesion 100, no. 8 (2024): 734–764, 10.1080/00218464.2023.2256670.

[61] G. Dou , M. Xu , Y. Hu , Y. Sun , H. Jiang , and G. Peng , “Improving the Mechanical Performance of Phase Change Microcapsule/Epoxy Composites via Alternately Enhancing Interface Bonding and Strength of Microcapsules,” Journal of Energy Storage 73 (2023): 109212, 10.1016/j.est.2023.109212.

[62] C. Fan , Z. Shan , G. Zou , L. Zhan , and D. Yan , “Interfacial Bonding Mechanism and Mechanical Performance of Continuous Fiber Reinforced Composites in Additive Manufacturing,” Chinese Journal of Mechanical Engineering 34, no. 1 (2021): 21, 10.1186/s10033-021-00538-7.

[63] C. A. Navarro , C. R. Giffin , B. Zhang , Z. Yu , S. R. Nutt , and T. J. Williams , “A Structural Chemistry Look at Composites Recycling,” Materials Horizons 7, no. 10 (2020): 2479–2486, 10.1039/D0MH01085E.

[64] A. Toldy , D. I. Poór , N. Geier , and Á. Pomázi , “Recent Advances and Challenges in the Mechanical and Chemical Recycling of Vitrimers and Fibre‐reinforced Vitrimer Composites: a Review,” Composites Part B: Engineering 306 (2025): 112760, 10.1016/j.compositesb.2025.112760.

[65] T. Granath , P. Löbmann , and K. Mandel , “Oxidative Precipitation as a Versatile Method to Obtain Ferromagnetic Fe_3_O_4_ Nano‐ and Mesocrystals Adjustable in Morphology and Magnetic Properties,” Particle & Particle Systems Characterization 38, no. 3 (2021): 2000307.

[66] S. Aravind , S. P. Sivapirakasam , K. R. Balasubramanian , and M. Surianarayanan , “Thermo‐kinetic Studies on Azodicarbonamide/Potassium Periodate Airbag Gas Generants,” Process Safety and Environmental Protection 144 (2020): 15–22, 10.1016/j.psep.2020.06.050.

[67] H. Jaafar and G. Sims , “The Thermal Decomposition of Azodicarbonamide (ADC),” Cellular Polymers 12, no. 4 (1993): 303–316.

[68] I. K. Abdel Maksoud , G. Bassioni , N. Nady , S. A. Younis , M. M. Ghobashy , and M. S. A. Abdel‐Mottaleb , “Radiation Synthesis and Chemical Modifications of p(AAm‐co‐AAc) Hydrogel for Improving Their Adsorptive Removal of Metal Ions From Polluted Water,” Scientific Reports 13, no. 1 (2023): 21879, 10.1038/s41598-023-49009-0. PMC10710999 38072847

[69] Á. Kmetty , K. Litauszki , and D. Réti , “Characterization of Different Chemical Blowing Agents and Their Applicability to Produce Poly(Lactic Acid) Foams by Extrusion,” Applied Sciences 8, no. 10 (2018): 1960, 10.3390/app8101960.

[70] M. F. Haque , S. Park , and D. Yang , “Gas Sensors in Harsh Environments: Challenges and Advances in High Temperature, High Humidity, Radiative and Corrosive Conditions,” Journal of Science: Advanced Materials and Devices 10, no. 4 (2025): 101049.

[71] M. Chen , X. Chen , S. Ray , and K. Yam , “Stabilization and Controlled Release of Gaseous/Volatile Active Compounds to Improve Safety and Quality of Fresh Produce,” Trends in Food Science & Technology 95 (2020): 33–44, 10.1016/j.tifs.2019.11.005.

[72] Z. Yuan , J. Ding , Y. Zhang , et al., “Components, Mechanisms and Applications of Stimuli‐responsive Polymer Gels,” European Polymer Journal 177 (2022): 111473, 10.1016/j.eurpolymj.2022.111473.

[73] M. S. Harris , J. A. Foster , and A. J. Ryan , “Reducing Gas‐loss in Rigid Polyisocyanurate Foams Using Metal–organic Frameworks,” Materials Advances 6, no. 23 (2025): 9125–9132, 10.1039/D5MA01002K.

[74] J. B. West , “The Original Presentation of Boyle's Law,” Journal of Applied Physiology 87, no. 4 (1999): 1543–1545, 10.1152/jappl.1999.87.4.1543. 10517789

[75] J. D. W. Madden , N. A. Vandesteeg , P. A. Anquetil , et al., “Artificial Muscle Technology: Physical Principles and Naval Prospects,” IEEE Journal of Oceanic Engineering 29, no. 3 (2004): 706–728, 10.1109/JOE.2004.833135.

[76] V. H. Ebron , Z. Yang , D. J. Seyer , et al., “Fuel‐powered Artificial Muscles,” Science 311, no. 5767 (2006): 1580–1583, 10.1126/science.1120182. 16543453

[77] S.‐D. Gravert , E. Varini , A. Kazemipour , et al., “Low‐voltage Electrohydraulic Actuators for Untethered Robotics,” Science Advances 10, no. 1 (2024): adi9319, 10.1126/sciadv.adi9319. PMC10775996 38181082

[78] H. Yang , G. Wei , L. Ren , L. Yan , and D. Caldwell , “Compliant Actuators That Mimic Biological Muscle Performance With Applications in a Highly Biomimetic Robotic Arm,” Advanced Bionics 1, no. 1 (2025): 2–18, 10.1016/j.abs.2024.12.001.

[79] S. M. Mirvakili , E. Haghighat , and D. Sim , “Machine Learning‐Enabled Precision Position Control and Thermal Regulation in Advanced Thermal Actuators,” Advanced Materials Technologies 9, no. 9 (2024): 2301769, 10.1002/admt.202301769.

[80] M. Acerce , E. K. Akdoğan , and M. Chhowalla , “Metallic Molybdenum Disulfide Nanosheet‐based Electrochemical Actuators,” Nature 549, no. 7672 (2017): 370–373, 10.1038/nature23668. 28854166

[81] A. Miriyev , K. Stack , and H. Lipson , “Soft Material for Soft Actuators,” Nature Communications 8, no. 1 (2017): 596, 10.1038/s41467-017-00685-3. PMC5605691 28928384

[82] S. Kim , S.‐W. Lee , H. Kwon , et al., “Soft Magnetic Artificial Muscles With High Work Density and Actuation Strain via Dual Cross‐Linking Design,” Advanced Functional Materials 36, no. 3 (2026): 16218, 10.1002/adfm.202516218.

[83] C. Lamuta , S. Messelot , and S. Tawfick , “Theory of the Tensile Actuation of Fiber Reinforced Coiled Muscles,” Smart Materials and Structures 27, no. 5 (2018): 055018, 10.1088/1361-665X/aab52b.

[84] V. Nguyen , B. Lilly , and C. Castro , “The Exoskeletal Structure and Tensile Loading Behavior of an Ant Neck Joint,” Journal of Biomechanics 47, no. 2 (2014): 497–504, 10.1016/j.jbiomech.2013.10.053. 24287400

[85] R.‐L. Guo , S.‐H. Liu , and Y.‐M. Lu , “Investigation of How Pressure Influences the Thermal Decomposition Behavior of Azodicarbonamide,” Journal of Loss Prevention in the Process Industries 83 (2023): 105062, 10.1016/j.jlp.2023.105062.

[86] J. A. Reyes‐Labarta and A. Marcilla , “Kinetic Study of the Decompositions Involved in the Thermal Degradation of Commercial Azodicarbonamide,” Journal of Applied Polymer Science 107, no. 1 (2008): 339–346, 10.1002/app.26922.

[87] J. Lobos , S. P. Thirumuruganandham , and M. A. Rodríguez‐Pérez , “Density Gradients, Cellular Structure and Thermal Conductivity of High‐Density Polyethylene Foams by Different Amounts of Chemical Blowing Agent,” Polymers 14, no. 19 (2022): 4082, 10.3390/polym14194082. PMC9573033 36236030

[88] Z. Ping , Q. Nguyen , S. Chen , J. Zhou , and Y. Ding , “States of Water in Different Hydrophilic Polymers — DSC and FTIR Studies,” Polymer 42, no. 20 (2001): 8461–8467.

[89] S. Arai and M. Doi , “Skin Formation and Bubble Growth During Drying Process of Polymer Solution,” European Physical Journal E 35, no. 7 (2012): 57. 10.1140/epje/i2012-12057-2 22772595

[90] C. Ajith , A. P. Deshpande , and S. Varughese , “Effect of Crosslink‐induced Heterogeneities on the Transport and Deformation Behavior of Hydrophilic Ionic Polymer Membranes,” Polymer Journal 54, no. 2 (2022): 179–190, 10.1038/s41428-021-00546-3.

[91] M. H. Rich , M. K. Lee , N. Marshall , et al., “Water–Hydrogel Binding Affinity Modulates Freeze‐Drying‐Induced Micropore Architecture and Skeletal Myotube Formation,” Biomacromolecules 16, no. 8 (2015): 2255–2264, 10.1021/acs.biomac.5b00652. 26113238

[92] T. Tanaka and D. J. Fillmore , “Kinetics of Swelling of Gels,” The Journal of Chemical Physics 70, no. 3 (1979): 1214–1218, 10.1063/1.437602.

[93] N. A. Peppas and E. W. Merrill , “Crosslinked Poly(vinyl alcohol) Hydrogels as Swollen Elastic Networks,” Journal of Applied Polymer Science 21, no. 7 (1977): 1763–1770, 10.1002/app.1977.070210704.

[94] K. K. Innes , “Temperature Dependence of Viscosity of Liquids,” The Journal of Physical Chemistry 60, no. 6 (1956): 817–818, 10.1021/j150540a039.

[95] G. Macetti , L. Sironi , C. Rovida , I. Geremia , R. Soave , and L. L. Presti , “On the Solubility of Azodicarbonamide in Water/DMSO Mixtures: An Experimental and Computational Study,” Royal Society Open Science 11, no. 6 (2024): 231831, 10.1098/rsos.231831. PMC11296043 39100163

[96] H.‐S. Park and X.‐P. Dang , “Reduction of Heat Losses for the In‐line Induction Heating System by Optimization of Thermal Insulation,” International Journal of Precision Engineering and Manufacturing 14, no. 6 (2013): 903–909, 10.1007/s12541-013-0119-6.

[97] S. Yu , H. Zhang , Z. Liu , et al., “Design and Optimization of Electromagnetic Induction Heating Pyrolysis Reactor Based on Load Impedance Model and Heat Transfer Model,” Chemical Engineering Journal 509 (2025): 160851, 10.1016/j.cej.2025.160851.

[98] M. Pensek , N. Holecek , H. Gjerkes , and I. Golobic , “Energy Consumption Analysis of Domestic Oven,” Journal of Mechanical Engineering 51, no. 7‐8 (2004): 405–410.

[99] H. Memon , Y. Wei , and C. Zhu , “Recyclable and Reformable Epoxy Resins Based on Dynamic Covalent Bonds—Present, Past, and Future,” Polymer Testing 105 (2022): 107420, 10.1016/j.polymertesting.2021.107420.

[100] Y. Feng , Z. Zhang , D. Yue , et al., “Recent Progress in Degradation and Recycling of Epoxy Resin,” Journal of Materials Research and Technology 32 (2024): 2891–2912, 10.1016/j.jmrt.2024.08.095.

[101] X. Zhao , Y. Long , S. Xu , X. Liu , L. Chen , and Y.‐Z. Wang , “Recovery of Epoxy Thermosets and Their Composites,” Materials Today 64 (2023): 72–97.

[102] I. Dey and S. Bose , “Vitrimers: Bridging the Recycling Gap Between Thermosets and Thermoplastics,” Journal of Materials Science: Composites 7, no. 1 (2026): 1.

[103] M. Hayashi and R. G. Ricarte , “Towards the Next Development of Vitrimers: Recent Key Topics for the Practical Application and Understanding of the Fundamental Physics,” Progress in Polymer Science 170 (2025): 102026.

[104] G. R. Jo , M. B. Yun , Y. Hun Son , et al., “Facile Synthesis of Epsilon Iron Oxides via Spray Drying for Millimeter‐wave Absorption,” Chemical Communications 58, no. 81 (2022): 11442–11445, 10.1039/D2CC03168J. 36148584

[105] W. C. Oliver and G. M. Pharr , “An Improved Technique for Determining Hardness and Elastic Modulus Using Load and Displacement Sensing Indentation Experiments,” Journal of Materials Research 7, no. 6 (1992): 1564–1583, 10.1557/JMR.1992.1564.

