# Supplementary material for: Inductively Expandable Supraparticles as Microscopic Force Generators for Remote Mechanical Actuation
Source: Adv Mater. 2026 Sep 15;38(61):e74932. doi: 10.1002/adma.74932 (PMC13726705; doi:10.1002/adma.74932)
Supplement: Supplementary file 1 [file ADMA-38-e74932-s004.docx]

# Supporting Information

**Inductively expandable supraparticles as microscopic force generators for remote mechanical actuation**

*Leoni Luthardt, Stephan Müssig, Robert Luxenhofer, and Karl Mandel^*^*

**Figure S1.** Characterization of the utilized magnetic NPs, octahedral FIONs (a1) and spherical ZnCo ferrite (ZnCoFe_2_O_4_) NPs (a2). ZnCo ferrite NPs exhibit a mean size of 20 nm, FIONs of 48 nm (b), while during induction heating, FIONs reach temperatures of 570 °C and ZnCo ferrites of 220 °C (c). Both types show a high magnetic moment between 80 and 90 emu g^-1^ with FIONs exhibiting a significantly larger coercivity (d).

**Figure S2.** Characterization of the utilized blowing agents. a) SEM of untreated ADCA (a1) and P(AAm-co-AAc) (a2). b) TGA of both blowing agents, showcasing decomposition for ADCA at 200 °C and that for P(AAm-co-AAc) starting around 250 °C.

**Figure S3.** Investigation of the functionality in the ADCA- (a) and P(AAm-co-AAc)-based (b) model systems after exposure to ambient conditions for one year. Heating curves are comparable to the pristine state (a1, b1), while gas generation (a2-3) and volume expansion (b2-3) is retained at the same conditions.

**Determination of the quantities given for the lifting experiments**

$$Pressure=p=p_{load}+p_{friction/syringe}=\frac{mg}{A}+0.275 bar$$

**(1)**

$$Displacement= \Delta V= V_{end}-V_{beginning}$$

**(2)**

$$Work output=W=p\Delta V$$

**(3)**

$$Energy conversion efficiency=\eta=\frac{W}{E_{input}}\cdot100$$

**(4)**

$$Power density =PD=\frac{W}{{t_{stroke}\cdot m}_{SP}}$$

**(5)**

In this, *V_end_* and *V_beginning_* signify the volume of the extended and compressed syringe before and after induction heating, *g* is the gravitational constant (9.81 m s^-2^), *m* the mass of the added weight on top of the syringe, *A* the area of the syringe bottom calculated from *πr^2^* (with *r* being 2.5 mm), *E_input_* the provided electrical energy of the induction heater, *I* the utilized current, and *t* the time of the induction heating pulse), *t_stroke_* the time until the syringe plunger is lifted (1 s for ADCA, 5 s for P(AAm-*co*-AAc)), and *m_SP_* the supraparticle mass within the syringe (20 mg). Note that the energy conversion efficiency was calculated for an induction heating period of 1 s for the ADCA-based system (corresponding to 0.6731 Wh) and 5 s for the P(AAm-*co*-AAc)-based system (corresponding to 2.787 Wh).

**Figure S4.** Chemical decomposition of ADCA in three steps, as showcased in literature.^[1,2]^

**Figure S5.** TGA of the ADCA:FION 1:1 model system before (light blue) and after (dark blue) induction heating. Remaining mass loss even after induction heating confirms residual solid byproducts from ADCA decomposition within the SP structure.

**Figure S6.** ATR-IR spectroscopy investigation of both model SP systems in different states. a) ADCA:FION 1:1 SP system shows a decrease of band intensity after induction heating as compared to the pristine state. ADCA decomposition is evidenced by comparing ATR-IR spectra before and after induction heating of the model system, due to the substantial decrease of band intensities succeeding heating, especially the sharp amide C=O stretching band at 1710 cm^‑1^. b) P(AAm-co-AAc):FION 8:2 SP system displays a characteristic O-H stretching vibration after humidity treatment, before losing characteristic bands after induction heating due to polymer decomposition processes.

**Figure S7.** MPS investigation of both model SP systems in different states. MPS presents a non-destructive way to distinguish before and after states, giving information of whether the trigger has already been performed. a) ADCA:FION 1:1 SP system shows an increase of the A5/A3 ratio upon the induction trigger. The MPS signal of the system changes upon the induction trigger, which is in accordance with a mechanical and structural rearrangement causing a change in the local distance between magnetic NPs, as would be expected after sudden gas release. As decomposed ADCA no longer works as a spacer between magnetic NPs, their interparticle coupling and dipolar interactions change presumably due to the closer distance.^[3,4]^ b) P(AAm-co-AAc):FION 8:2 SP system exhibits an increase in the A5/A3 ratio after humidity treatment and again after induction heating. As swelling of the superabsorber in the SP system brings about a decreased local viscosity compared to the dry state and therefore increases mobility of the magnetic NP component, the interparticle distance is increased, confirmed by an altered MPS signal

**Figure S8.** Comparison between ADCA:FION 1:1 systems spray-dried in DMSO and water, with both components dispersed in water (a), only ADCA dissolved in DMSO (b), and both parts dissolved/dispersed in DMSO (c), respectively. Water leads to precipitation of ADCA during spray-drying, while using purely DMSO brings a well intermixing of both components within the SP. While the inductively heated structures are comparable among all cases, the heat distribution is considered favorable in the pure DMSO system.

**Figure S9.** Investigation of optimal conditions for the humidity exposure of the P(AAm-co-AAc):FION model system (8:2). a) Variation of the relative humidity exposure at 4 h, with a1) showing an exponential increase of water uptake with increasing relative humidity up to 99%, corresponding to a water uptake of 44%. a2-7) SEM shows that lower relative humidities retain the original SP structure, while higher exposure levels transform the sample into a bulk like material. b) Variation of the exposure time at 98% relative humidity, with b1) showing a sharp increase of water uptake up to 8 h with levelling up to 24 h. b2-7) SEM displays that with higher exposure time, the original SP structure is continually lost and the material surface becomes smoother as more water is taken up into the structure. Please note, samples depicted in the panels a7 and b5 are identical and two different magnifications are shown.

**Figure S10.** Characterization of the ADCA:ZnCo ferrite 1:1 system. a) Vibrating sample magnetometer (VSM) measurements compared to the FION-based system, displaying a significantly reduced coercivity. b) The response time to the induction heating pulse is slower compared to FION-based systems, now taking 2.3 s instead of 0.5 s. c) SEM micrographs show well intermixed SPs in the pristine state (c1-2) that are converted to multi-micron networks upon induction heating (d1-2).

**Figure S11.** Characterization of the P(AAm-co-AAc):ZnCo ferrite 1:1 system. a) Vibrating sample magnetometer (VSM) measurements compared to the FION-based system, displaying a reduced coercivity. b) The response time to the induction heating pulse is slower compared to FION-based systems, now taking 3 s instead of 1 s for the initial response and almost 10 s instead of 3 s for full expansion. c) SEM micrographs show well intermixed SPs in the pristine state (c1-2) that are humidity-treated (d1-2), before pores are generated upon induction heating (d1-2).

**Figure S12.** Reversibility tests in P(AAm-co-AAc):magnetic NPs 8:2 systems. a) Set-up for the tests, consisting of spray-drying the corresponding components (a1) and treating them with humidity (a2), before conducting the first induction heating run for different exposure times (a3). The second humidity treatment is performed afterwards (a4) prior to the second induction heating run to probe whether functionality is maintained (a5). b) FION systems heated for 5 s in the first run display a successful trigger in the second run, retaining functionality. c) FION systems heated for 10 s during the first run only show a minor trigger in the second, indicating the onset of polymer decomposition during the first run. d) FION systems heated for 30 s in the first run exhibit no apparent trigger during the second run, hinting towards full polymer decomposition during the first run. e) In contrast, ZnCo ferrite systems show a successful trigger in the second run even after initial induction heating for 30 s, showcasing that the polymer is not decomposed due to the lower achieved maximum temperature, making reversibility feasible with this system.

**Figure S13.** Confirmation of ratios of blowing agent:FIONs between 1:1 and 9:1 with TGA (a1, a2) and VSM (b1, b2). In TGA, the mass loss corresponds approximately to the amount of blowing agent lost. In VSM, the mass-normalized magnetic moment is diminished for increasing content of blowing agent and decreasing FION content.

**Figure S14.** Induction heating curves for different ratios in a) ADCA:FION and b) P(AAm-co-AAc):FION systems. The latter are displayed for the humidity-treated and dried (b1), only humidity-treated (b2), and pristine state (b3). Higher blowing agent content results in lower initial heating rate and lower maximum reached temperatures. Pre-treatment of the P(AAm-co-AAc)-based system results in faster heating rates and trigger response.

**Discussion about the ratio variation in both systems**

Ratio variation in both systems brings about (quasi-)spherical SPs with intermixed components in every case, where the higher FION share is evidenced on the surface as seen in SEM imaging, respectively (see **Figure S15** and **Figure S16**). After induction heating, in the 9:1 ADCA:FION system, decomposition is not fully completed in the investigated time frame, which is also in line with the lower maximum reached temperatures. Additionally, lower ADCA contents result in a rather loose pore structures, while the dense macroporous FION network structures only form at higher shares, presumably because of faster ADCA decomposition with enlarged FION content that allows a fast pressure buildup (see **Figure S15**). Similarly, in the P(AAm-*co*-AAc)-based systems, swelling after humidity treatment is more pronounced for a higher superabsorber content, as more material for swelling is available overall. Following induction heating, typical large pores are also only visible for low FION ratios, as higher contents presumably initiate polymer decomposition faster than the desired trigger, in line with higher heating rates and temperatures during induction heating (see **Figure S16**).

**Figure S15.** SEM micrographs of systems with different ADCA:FION ratios, namely 1:1 (a), 6:4 (b), 7:3 (c), 8:2 (d), and 9:1 (e). In the pristine state, all systems display quasi-spherical SP morphologies with ADCA and FIONs well intermixed. In the inductively heated state, ADCA is not fully decomposed for its highest share in the investigated timeframe (e), while loose pore structures (c, d) become dense macroporous FION networks (a, b) with higher FION share, presumably due to the faster decomposition of ADCA and hence a faster pressure buildup.

**Figure S16.** SEM micrographs of systems with different P(AAm-co-AAc):FION ratios, namely 1:1 (a), 6:4 (b), 7:3 (c), 8:2 (d), and 9:1 (e). In the pristine state, all systems display spherical SP morphologies with FIONs uniformly embedded in the P(AAm-co-AAc) matrix. After humidity treatment, swelling increases for increasing superabsorber share. Following induction heating, typical large pores only visible for higher superabsorber contents, as at lower shares, polymer decomposition is presumably faster than triggering the desired effect. Please note, sample depicted in panel b3 is identical to sample depicted in Figure S9, panels a7 and b5, but shown at different magnifications.

**Table S1.** Calculation of volume displacement, expansion rate, and mass-normalized work output for induction heating experiments in a syringe for ratio variations of both inductively expandable systems. Note that the pressure was determined as the sum of atmospheric pressure and the friction between the syringe and piston, adding up to 1.29 bar. All values were obtained as an average of three individual experiments each. The actuation performance was maintained through all repetitions, with no reportable failure rates.

|  | Displacement / mL | Expansion rate | Work output / J g^-1^ |
| --- | --- | --- | --- |
| ADCA:FION | | | |
| 1:1 | 0.60 ± 0.11 | 12.3 ± 1.1 | 6.0 ± 1.3 |
| 6:4 | 0.55 ± 0.05 | 12.5 ± 1.6 | 7.0 ± 0.2 |
| 7:3 | 0.56 ± 0.02 | 11.6 ± 0.0 | 6.6 ± 0.7 |
| 8:2 | 0.60 ± 0.06 | 12.2 ± 1.4 | 8.3 ± 0.3 |
| 9:1 | 0.67 ± 0.11 | 15.4 ± 0.0 | 9.0 ± 0.2 |
| P(AAm-*co*-AAc):FION | | | |
| 1:1 | 0.6 ± 0.3 | 15 ± 8 | 2.3 ± 0.6 |
| 6:4 | 0.5 ± 0.2 | 13 ± 5 | 2.1 ± 0.5 |
| 7:3 | 0.24 ± 0.09 | 8 ± 2 | 1.6 ± 0.1 |
| 8:2 | 0.33 ± 0.15 | 11 ± 6 | 1.5 ± 0.2 |
| 9:1 | 0.29 ± 0.04 | 7.4 ± 1.5 | 0.9 ± 0.2 |

**Table S2.** Determination of onset times of the inductively triggered expansion in both SP systems, as determined from three individually performed induction heating experiments. All values are given in s.

| Ratio blowing agent:NPs | ADCA-based | P(AAm-*co*-AAc)-based – start | P(AAm-*co*-AAc)-based – full |
| --- | --- | --- | --- |
| 9:1 | 3.88 ± 0.05 | 3.28 ± 0.03 | 7.87 ± 0.08 |
| 8:2 | 1.40 ± 0.01 | 1.63 ± 0.10 | 3.7 ± 0.3 |
| 7:3 | 0.84 ± 0.05 | 1.25 ± 0.14 | 2.5 ± 0.3 |
| 6:4 | 0.63 ± 0.03 | 0.80 ± 0.02 | 1.15 ± 0.18 |
| 1:1 | 0.53 ± 0.05 | 0.53 ± 0.05 | 0.740 ± 0.02 |

**Table S3.** Calculation of the overall, mass-normalized, magnetic NP-normalized, and blowing agent-normalized material-only volume change of the ratio-varied P(AAm-co-AAc)-based systems, determined as the average of three individual induction heating experiments in a syringe each.

|  | Overall V change / mL | V change per total SP / mL mg^-1^ | V change per magnetic NP / mL mg^-1^ | V change per blowing agent / mL mg^-1^ |
| --- | --- | --- | --- | --- |
| 1:1 | 0.02 ± 0.01 | 0.6±0.1 · 10^-3^ | 0.3±0.1 · 10^-3^ | 0.3±0.1 · 10^-3^ |
| 6:4 | 0.03 ± 0.01 | 1.1±0.1 · 10^-3^ | 0.4±0.0 · 10^-3^ | 0.6±0.0 · 10^-3^ |
| 7:3 | 0.03 ± 0.01 | 1.6±0.5 · 10^-3^ | 0.6±0.2 · 10^-3^ | 1.1±0.4 · 10^-3^ |
| 8:2 | 0.05 ± 0.02 | 1.8±0.2 · 10^-3^ | 0.4±0.0 · 10^-3^ | 1.5±0.1 · 10^-3^ |
| 9:1 | 0.09 ± 0.02 | 2.2±0.5 · 10^-3^ | 0.2±0.1 · 10^-3^ | 2.0±0.5 · 10^-3^ |

**Discussion of content and structure variation in the P(AAm-*co*-AAc)-based system**

Adding NaHCO_3_ as gas-generating agent that decomposes at lower temperatures compared to water^[5]^ leads to a fine intermixing of all three SP components in the pristine state (see **Figure S17**). Upon induction heating, more pores are formed in the now sponge-like material, which results in a more extensive volume expansion, while the heating and trigger response behavior mostly remain unchanged. Apart from content variation, the SP structure itself can be modified from an intermixed to a core-shell architecture with a FION core and a P(AAm-*co*-AAc) shell (see **Figure S18**). In the humid state, this leads to FION SP cores dispersed in the swollen superabsorber matrix, inducing a more uniform heat generation compared to only NPs. As a result, induction heating produces significantly larger pores and a more extensive volume expansion, however on the cost of response times, as heat transport is delayed when the superabsorber is situated only on the outside of the SP, which is why full expansion takes almost one minute. Both methods – content and structure modification – are therefore useful design parameters to tune volume expansion and response time.

**Figure S17.** Characterization of a content-modified P(AAm-co-AAc)-based system. a) SP structure consisting of intermixed FIONs, P(AAm-co-AAc), and NaHCO_3_. b) Induction heating in 20 s leading to optimized volume expansion. c) SEM micrographs showing an intermixed SP structure in the pristine state, while all components are still intermixed in the humidity-treated state (d). e) After induction heating, a sponge-like porous structure is formed due to the additional gas-generating component. f) Induction heating curves of systems with constant FION content with and without the addition of NaHCO_3_ show similar behavior, just like the trigger response times (g).

**Figure S18.** Characterization of a structure-modified P(AAm-co-AAc)-based system. a) SP structure consisting of a FION core and a P(AAm-co-AAc) shell. b) Induction heating in 50 s leading to optimized volume expansion. c1) SEM micrographs showing a P(AAm-co-AAc) coated FION core in the pristine state, with the FION SP cores dispersed in the superabsorber matrix in the swollen state (c2). c3) After induction heating, large pores are created with FION cores still apparent in the polymer residue. d) Induction heating curves of differently structured systems showing significantly slower heating for the core-shell structure, together with decelerated full expansion (e).

**Figure S19.** Oven treatment of inductively triggerable SP systems prior to induction heating. a) With the ADCA:FION 1:1 system, functionality is retained after oven heating at 100 °C for 1 h, limited at 150 °C (b), and canceled out at 200 °C (c), due to the decomposition of ADCA within the SP structure at higher temperatures. d-f) For the P(AAm-co-AAc):FION 8:2 system, functionality is retained even after oven heating at 200 °C for 1 h, as external heating dries the polymer shell and therefore contributes to the working mechanism.

**Figure S20.** Importance of the heating rate of magnetic NPs to achieve the desired functionality. a) Comparison between slow (a1) and fast (a2) heating rate. Slow heating rates lead to low gas production and high dissipation, which prevents pressure buildup, while high heating rates bring high and fast gas generation, leading to pressure development. b) Using CoFe_2_O_4_ NPs in the ADCA-based systems lead to a temporally delayed trigger (b1) with a weaker effect (b2, b3) due to gas dissipation. c) Using CoFe_2_O_4_ NPs in the P(AAm-co-AAc)-based system leads to enormous heat and gas dissipation, resulting in no trigger under these conditions (c2, c3).

**Figure S21.** Characterization of epoxy opening experiments by inductively heating both ADCA- and P(AAm-co-AAc)-based systems. a) All epoxy opening experiments were repeated four times for each system, revealing gas accumulation prior to cracking for ADCA-based materials (a1) and direct matrix disruption by rapid particle expansion for P(AAm-co-AAc)-based systems (a2). b) Optical microscopy analysis of inductively opened epoxy resins reveal cracks for both systems, with a smaller width for the ADCA-based system (b1) and wider openings for P(AAm-co-AAc)-based materials, revealing physically expanded particles inside (b2).

**Table S4.** Exemplary Berkovich measurements of the epoxy resin inductively opened by ADCA:FION 1:1 SPs at the outside of the epoxy sample and near the crack in the middle, respectively.

|  | ADCA:FION 1:1  Outside | ADCA:FION 1:1  Near crack |
| --- | --- | --- |
| Modulus / GPa | 3.60 ± 0.00 | 3.20 ± 0.10 |
| Hardness /GPa | 0.18 ± 0.01 | 0.15 ± 0.01 |
| Drift correction / nm s^-1^ | -0.68 ± 0.09 | -0.79 ± 0.03 |

**References**

[1]	J. A. Reyes-Labarta, A. Marcilla, "Kinetic study of the decompositions involved in the thermal degradation of commercial azodicarbonamide", *Journal of Applied Polymer Science* **2008**, *107* (1), 339–346.

[2]	R.-L. Guo, S.-H. Liu, Y.-M. Lu, "Investigation of how pressure influences the thermal decomposition behavior of azodicarbonamide", *Journal of Loss Prevention in the Process Industries* **2023**, *83*, 105062.

[3]	A. Wolf, M. Heinlein, N. Kent, S. Müssig, K. Mandel, "Bulk Magnetic Properties Arise from Micron-Sized Supraparticle Interactions and Can be Modified on the Nanoscale", *Small* **2025**, *21* (13), e2412311.

[4]	S. Müssig, F. Fidler, D. Haddad, K.-H. Hiller, S. Wintzheimer, K. Mandel, "Supraparticles with a Magnetic Fingerprint Readable by Magnetic Particle Spectroscopy: An Alternative beyond Optical Tracers", *Advanced Materials Technologies* **2019**, *4* (9).

[5]	T. C. Keener, G. C. Frazier, W. T. Davis, "Thermal decomposition of sodium bicarbonate", *Chemical Engineering Communications* **1985**, *33* (1-4), 93–105.
